# Effectiveness of inactivated influenza vaccine in autoimmune rheumatic diseases treated with disease-modifying anti-rheumatic drugs

**DOI:** 10.1093/rheumatology/keaa078

**Published:** 2020-03-11

**Authors:** Georgina Nakafero, Matthew J Grainge, Puja R Myles, Christian D Mallen, Weiya Zhang, Michael Doherty, Jonathan S Nguyen-Van-Tam, Abhishek Abhishek

**Affiliations:** k1 Academic Rheumatology, School of Medicine, University of Nottingham, Nottingham; k2 Epidemiology and Public Health, School of Medicine, University of Nottingham, Nottingham; k3 Primary Care Centre Versus Arthritis, Keele University, Keele; k4 Nottingham NIHR Biomedical Research Centre, Nottingham, UK

**Keywords:** flu vaccine, autoimmune rheumatic diseases, mortality, infection

## Abstract

**Objectives:**

The effectiveness of inactivated influenza vaccine in people with autoimmune rheumatic disease (AIRDs) is not known. We investigated whether the influenza vaccine is effective in preventing respiratory morbidity, mortality and all-cause mortality in AIRD patients.

**Methods:**

Adults with AIRDs treated with DMARDs prior to 1 September of each year between 2006 and 2009, and 2010 and 2015 were identified from the Clinical Practice Research Datalink. Exposure and outcome data were extracted. Data from multiple seasons were pooled. Propensity score (PS) for vaccination was calculated. Cox-proportional hazard ratios (HRs) and 95% CIs were calculated, and were (i) adjusted, (ii) matched for PS for vaccination.

**Results:**

Data for 30 788 AIRD patients (65.7% female, 75.5% with RA, 61.1% prescribed MTX) contributing 125 034 influenza cycles were included. Vaccination reduced risk of influenza-like illness [adjusted HR (aHR) 0.70], hospitalization for pneumonia (aHR 0.61) and chronic obstructive pulmonary disease exacerbations (aHR 0.67), and death due to pneumonia (aHR 0.56) on PS-adjusted analysis in the influenza active periods (IAPs). The associations were of similar magnitude and remained statistically significant on PS-matched analysis except for protection from influenza-like illness, which became non-significant. Sub-analysis restricted to pre-IAP, IAP and post-IAP did not yield evidence of residual confounding on influenza-like illness and death due to pneumonia. Vaccination reduced risk of all-cause mortality, although IAP-restricted analysis demonstrated residual confounding for this outcome.

**Conclusion:**

Influenza vaccine associates with reduced risk of respiratory morbidity and mortality in people with AIRDs. These findings call for active promotion of seasonal influenza vaccination in immunosuppressed people with AIRDs by healthcare professionals.

**Rheumatology key messages**

- Inactivated influenza vaccine reduces influenza-like illness, hospitalization for pneumonia/chronic obstructive pulmonary disease exacerbation and death due to pneumonia in autoimmune rheumatic diseases.
- Inactivated influenza vaccine reduced all-cause mortality, but this could be due to residual confounding.
- Inactivated influenza vaccine should be actively promoted in people with autoimmune rheumatic diseases.

## Introduction

Influenza causes 291 000–650 000 deaths/year globally. It is estimated to cause 3.1 million hospitalized days and 31.4 million outpatient visits, costing 87.1 billion dollars to the USA economy annually [1, 2]. Inactivated influenza vaccine (IIV) prevents influenza and its complications, with the degree of protection depending on vaccine match [3, 4]. According to the latest Cochrane reviews, IIV prevents influenza and influenza-like illness (ILI) in adults ≥65 years old (risk ratio 0.42 and 0.59, respectively) and in young adults (risk ratio 0.41 and 0.84, respectively) [3, 5]. It prevents chronic obstructive pulmonary disease (COPD) exacerbation and lower respiratory tract infection (LRTI) in people with haematological malignancy [6, 7]. However, its efficacy remains unproven in asthma, cystic fibrosis and in healthcare workers for preventing influenza among care-home residents [8–10]. Although observational studies report that IIV prevents pneumonia, hospitalization and death, there is a paucity of randomized controlled trial (RCT) evidence for these outcomes [11–13].

Autoimmune rheumatic diseases (AIRDs) such as RA and SpA associate with increased risk of influenza and its complications, and seasonal flu vaccination is recommended annually [14, 15]. While the magnitude of serological response to IIV has been examined in AIRDs, its effectiveness in preventing patient-centred outcomes such as influenza, pneumonia and death have not been studied [16–18]. The only study to evaluate the effectiveness of IIV in the context of immunosuppression had <5% AIRD cases [11]. Moreover, there are concerns that MTX, the first-choice DMARD, and rituximab impair the serological response to IIV [16–18]. As lack of knowledge about the need for vaccination and vaccine effectiveness (VE) are barriers to vaccination, it is important to examine whether IIV prevents respiratory morbidity and mortality in AIRDs [19, 20]. Thus, the objectives of this study were to assess the effectiveness of IIV in preventing ILI, LRTI, pneumonia, COPD exacerbations and death in immunosuppressed AIRD patients.

## Methods

### Data source

Data from the Clinical Practice Research Datalink (CPRD) were used. Incepted in 1987, CPRD is a longitudinal anonymized electronic database containing health records of >10 million people in the UK [21]. CPRD participants are representative of the UK population in terms of age, sex and ethnicity [21]. It contains details of diagnoses stored as Read codes, a coded thesaurus of clinical terms, primary care prescriptions and immunizations. The data are enhanced by linkage with hospitalization (Hospital Episode Statistics) and mortality records (Office of National Statistics). Data on prescription of biologic agents prescribed by hospital-based rheumatologists are not recorded. Ethical approval was obtained from the Independent Scientific Advisory Committe (ISAC) of the Medicines and Healthcare Products Regulatory Agency, London (Ref: 16_288R).

### Study design: cohort study

#### Population

One or more Read code for RA, lupus or SpA (defined as either PsA, reactive arthritis, IBD-associated arthritis or AS) in receipt of one or more prescription of immunosuppressive DMARDs (MTX, leflunomide, sulfasalazine, AZA, ciclosporin, MMF or tacrolimus), registered in general practitioner (GP) surgeries validated to meet CPRD data standards, and ≥18 years in age. Participants only ever prescribed the immunomodulatory drug HCQ were excluded, as IIV is not recommended for HCQ prescription in the UK [22].

#### Annual cohorts

The study period (1 September 2006 to 31August 2016) was partitioned into influenza cycles of 12 months, beginning on 1 September of one year and ending on 31 August of the subsequent year. Data for the 2009–10 cycle were excluded due to the use of monovalent pandemic vaccine alongside trivalent IIV, and the almost complete dominance of pandemic influenza A(H1N1)pdm09 in the community.

To be included in a cohort for one influenza cycle, participants had to be contributing data on the 1 September, with at least 3 months’ registration at their current GP surgery, and receive a DMARD prescription in this period. The 3 months prior registration period was to allow the GP time to incorporate new patients into local at-risk registers and to invite them for vaccination. A subset of participants treated with long-term oral CS, defined as two or more CS prescriptions in this 3-month period was constructed for examining VE in those prescribed DMARDs and CS.

#### Exposure

IIV administration was the exposure of interest. Vaccination and date of vaccination were ascertained using Read codes and event date [23] (supplementary Table S1, available at *Rheumatology* online). An individual was defined as exposed (fully immunized) 14 days after vaccination, in keeping with the typical time taken for mounting a serological response to the IIV [22]. Influenza cycles in which the IIV was recorded in the CPRD as being administered elsewhere, e.g. at work or in community pharmacies, were excluded, as the exact date of vaccination is not available in the CPRD.

#### Outcomes

We included a range of serious, e.g. hospitalization and death, and less-serious outcomes, e.g. primary care consultation for respiratory infections.

Primary care consultation for LRTI was defined as diagnostic Read code for LRTI and antibiotic prescription on the same date without preceding antibiotic prescriptions or Read codes for LRTI within 21 days prior to the eligible LRTI diagnosis date. Published Read code lists were supplemented with additional codes as required (supplementary Table S1, available at Rheumatology online) [24].

Primary care consultation for ILI was defined as above using published Read codes supplemented with additional codes as above (supplementary Table S1, available at *Rheumatology* online) [25].

Primary care consultation for COPD exacerbation was defined as either Read code for acute COPD exacerbation, or oral prednisolone and oral antibiotic prescriptions occurring on the same date, with oral prednisolone not prescribed as maintenance treatment in people with COPD [24, 26]. Oral prednisolone was considered as maintenance if two or more prescriptions were issued within the previous 60 days.

Hospital admissions for pneumonia or COPD exacerbation were defined using International Classification of Diseases-10 codes in the inpatient Hospital Episode Statistics dataset.

Death including causes was defined using International Classification of Diseases-10 codes in the Office of National Statistics dataset.

#### Follow-up

In each of the influenza cycles, participants were followed-up from 1 September until the earliest of outcome date, date of death, date of last data collection, transfer out of the GP-surgery or 31 August of the following year. A participant was defined as having an outcome of interest on the date of first allocation of Read code or International Classification of Diseases-10 code in an influenza cycle.

IIV is most likely to have an effect on outcomes during periods of influenza virus circulation, and the proportion of ILI and other outcomes attributable to influenza virus activity is greatest. Therefore, we undertook additional analyses, restricting the outcomes to the influenza active period (IAP).

#### Propensity score

As participants at risk of influenza are more likely to be vaccinated, a propensity score (PS) for vaccination was calculated [12]. The PS included factors that account for confounding by indication [age, sex, socioeconomic status, smoking status (a dummy category was created for missing smoking data), at-risk conditions (i.e. chronic respiratory, heart, kidney or neurological disease, immunosuppression or diabetes), Charlson comorbidity index], and health-seeking behaviour (previous pneumococcal and influenza vaccination, and number of primary care consultations, number of prescribed drugs and number of hospital admissions in the 12 months prior to the 1 September of each year) [12]. A separate PS was calculated for each influenza cycle for every participant using logistic regression, treating vaccination status as the dependent variable.

### Statistical analyses

Data for all influenza cycles from every participant were included in a single dataset. Mean, (s.d.), *n* (%) and standardized difference (*d*) were used to examine covariate balance between exposed and unexposed. Cox regression was used to calculate hazard ratios (HRs) and 95% CIs with vaccination as exposure of interest, adjusted for calendar year, and included participant identifier as a clustering term to account for within-person correlation. Adjusted vaccine effectiveness (aVE) was calculated as (1 – adjusted HR) × 100. Different analyses were performed to evaluate the consistency of results across different methods of accounting for propensity for vaccination as follows.

#### PS-adjusted

Vaccination was a time-varying exposure, i.e. the time period from day-14 post-vaccination up to end of the influenza cycle was defined as exposed, whilst the period before this was classed as unexposed. Participants without a vaccination record in the influenza cycle were considered unexposed for the entire duration. PS distribution in the vaccinated and unvaccinated groups was plotted on a histogram (supplementary Fig. S1, available at *Rheumatology* online). To remove unexpected treatment effects, influenza cycles in which there was no vaccination in the highest decile of PS for vaccination, and vaccination in the lowest decile of PS for vaccination were excluded [27]. Sensitivity and subgroup analyses were performed, restricted to people with RA, excluding influenza cycles with mild immunosuppressive SSZ prescription alone, stratifying by age, and restricting the analysis to those additionally prescribed CS. Inverse-probability of treatment weighing (IPTW) using the PS was performed as an additional *post hoc* sensitivity analysis because covariate adjustment using PS potentially biases the estimation of marginal and conditional HRs in a time-to-event analysis [28].

#### PS-matched

A 1:1 matched cohort was constructed using greedy nearest neighbour matching without replacement specifying a maximum calliper width of 0.001. The unexposed participants were allocated a pseudo-exposure date of their matched exposed pair. Standardized difference (*d*) was used to examine covariate balance between the exposed and unexposed participants (see supplementary methods, available at *Rheumatology* online). Any covariates for which there was imbalance, defined as *d *>* *0.10, were included as additional covariates in the model [29]. Analyses for VE were stratified for pre-IAP, IAP and post-IAP to assess residual confounding. IAPs were defined as per Public Health England reports using information about rates of consultation for ILI, and isolation of the seasonal influenza virus from virological sentinel surveys. Data management and analysis were performed in Stata v14, Stata Corp LLC, Texas, USA.

## Results

Data for 30 788 participants, 65.66% female, 75.49% with RA, contributing 125 034 influenza cycles (87 212 vaccinated) were included (supplementary Table S2, available at *Rheumatology* online). During the follow-up period, there were mean (s.d.) 3.78 (2.46) vaccinations administered. A total of 15 355 participants (49.87%) received all possible vaccination, whereas 8444 (27.43%) missed more than one potential vaccination (supplementary Table S8, available at *Rheumatology* online). Some 2942 participants had COPD and contributed 9909 influenza cycles. There were 17 876 vaccinated influenza cycles PS-matched 1:1 to unprotected influenza cycles. The covariate imbalance between exposed and unexposed influenza cycles reduced following PS-matching (Table 1).

**Table 1:** Covariate balance before and after PS-matching

	Entire cohort			PS-matched sample		
	Vaccinated (*n* = 87 212)	Unvaccinated (*n* = 37 822)	*d* ^a^	Vaccinated (*n* = 17 876)	Unvaccinated (*n* = 17 876)	*d* ^a^
Continuous covariates, mean (s.d.)						
Age (years)	64.13 (12.76)	55.88 (13.64)	0.625	58.69 (13.39)	58.06 (13.92)	0.047
Charlson’s comorbidity index	1.34 (1.58)	0.75 (1.23)	0.415	1.16 (1.57)	0.92 (1.37)	0.162
Index of Multiple Deprivation	3.12 (1.42)	3.16 (1.41)	−0.032	3.15 (1.41)	3.16 (1.41)	−0.004
Number of prescriptions^b^	3.01 (7.13)	2.81 (4.88)	0.034	2.95 (9.33)	2.83 (4.78)	0.015
Number of consultations^b^	19.92 (12.35)	15.39 (10.91)	0.389	17.24 (11.81)	17.08 (11.37)	0.014
Number of hospitalizations^b^	0.15 (0.62)	0.12 (0.57)	0.040	0.19 (0.72)	0.13 (0.57)	0.100
Categorical covariates, *n* (%)						
Male	28 495 (32.67)	13 430 (35.51)	−0.060	6559 (36.69)	5894 (32.97)	0.078
Home visit	709 (0.81)	186 (0.49)	0.030	180 (1.01)	95 (0.53)	0.055
Current smoking	13 300 (15.25)	8656 (22.89)	−0.195	4018 (22.48)	3807 (21.30)	0.029
Previous influenza vaccination	77 419 (88.77)	8787 (23.23)	1.758	8608 (48.15)	8609 (48.16)	−0.0002
Previous pneumococcal vaccination	62 998 (72.24)	9214 (24.36)	1.092	7520 (42.07)	7327 (40.99)	0.022
Diabetes	10 509 (12.05)	1805 (4.77)	0.265	1786 (9.99)	1197 (6.70)	0.119
Immunosuppression	921 (1.06)	305 (0.81)	0.026	241 (1.35)	150 (0.84)	0.049
Chronic kidney disease	12 854 (14.74)	2780 (7.35)	0.237	2276 (12.73)	1634 (9.14)	0.115
Chronic respiratory disease	19 121 (21.92)	5205 (13.76)	0.214	3430 (19.19)	2986 (16.70)	0.065
Chronic heart disease	8208 (9.41)	1292 (3.42)	0.246	1352 (7.56)	863 (4.83)	0.113
Asplenia	34 (0.04)	26 (0.07)	−0.013	11 (0.06)	7 (0.04)	0.009

a Standardized difference.

b In previous 12 months. PS: propensity score.

### PS-adjusted analysis

IIV reduced risk of hospitalization for pneumonia, COPD exacerbation, all-cause mortality and death due to pneumonia (Table 2). On restricting follow-up period to IAPs, IIV reduced risk of primary care consultations for ILI (Table 3). These associations remained significant on trimming-tails (Tables 2 and 3).

**Table 2:** Influenza vaccine effectiveness in AIRDs using data from entire influenza cycle: propensity score-adjusted analysis

Outcomes	Vaccinated	Event rate (95% CI)/1000 person-years	Unadjusted HR (95% CI)	Adjusted HR^a^ (95% CI)	Adjusted HR^b^ (95% CI)	Adjusted VE^c^ % (95% CI)
Primary care consultation for LRTI requiring antibiotics	No	78.02 (75.59, 80.53)	1.00	1.00	1.00	–
	Yes	100.97 (98.53, 103.46)	1.41 (1.34, 1.49)	1.07 (1.01, 1.13)	1.04 (0.97, 1.11)	−4 (−11, 3)
Primary care consultation for ILI	No	6.96 (6.27, 7.73)	1.00	1.00	1.00	–
	Yes	7.09 (6.48, 7.75)	0.91 (0.78, 1.06)	0.91 (0.74, 1.12)	0.89 (0.70, 1.13)	−11 (−13, 30)
Primary care consultation for COPD exacerbation	No	240.89 (222.47, 260.84)	1.00	1.00	1.00	−
	Yes	275.45 (262.09, 289.48)	1.09 (0.94, 1.27)	0.96 (0.83, 1.12)	0.93 (0.79, 1.10)	7 (−21, 10)
Hospitalization for pneumonia	No	17.03 (15.59, 18.60)	1.00	**1.00**	**1.00**	–
	Yes	20.37 (18.98, 21.86)	1.29 (1.12, 1.48)	**0.59 (0.51**, **0.69)**	**0.69 (0.58**, **0.83)**	**31 (17**, **42)**
Hospitalization for COPD exacerbation	No	109.42 (93.85, 127.58)	1.00	**1.00**	**1.00**	–
	Yes	88.74 (79.46, 99.10)	0.78 (0.57, 1.05)	**0.59 (0.44, 0.80)**	**0.65 (0.46**, **0.93)**	**35 (7**, **54)**
All-cause death	No	18.75 (17.59, 19.99)	1.00	**1.00**	**1.00**	**–**
	Yes	21.77 (20.69, 22.91)	1.10 (1.00, 1.20)	**0.52 (0.47**, **0.59)**	**0.66 (0.57**, **0.75)**	**34 (25**, **43)**
Deaths due to pneumonia	No	5.33 (4.56, 6.24)	1.00	**1.00**	**1.00**	**–**
	Yes	5.74 (5.03, 6.56)	1.08 (0.85, 1.35)	**0.47 (0.35**, **0.63)**	**0.67 (0.47**, **0.96)**	**33 (53**, **4)**

a Model 1: adjusted for propensity score for IIV and year.

b Model 2: adjusted for propensity score for IIV and year with PS trimmed tails.

c VE: vaccine effectiveness from Model 2. AIRD: autoimmune rheumatic disease; COPD: chronic obstructive pulmonary disease; HR: hazard ratio; ILI: influenza-like illness; LRTI: lower respiratory tract infection. Significant results are in bold.

**Table 3:** Influenza vaccine effectiveness in AIRDs restricted to influenza-active periods: propensity score-adjusted analysis

Outcomes	Vaccinated	Event rate (95% CI)/1000 person-years	Unadjusted HR (95% CI)	Adjusted HR^a^ (95% CI)	Adjusted HR^b^ (95% CI)	Adjusted VE^c^ % (95% CI)
Primary care consultation for LRTI requiring antibiotics	No	86.17 (82.53, 89.96)	1.00	1.00	1.00	–
	Yes	114.43 (111.30, 117.65)	1.38 (1.30, 1.46)	1.05 (0.98, 1.13)	1.04 (0.96, 1.13)	−4 (−13, 4)
Primary care consultation for ILI	No	9.23 (8.12, 10.51)	1.00	1.00	1.00	–
	Yes	7.99 (7.21, 8.85)	0.85 (0.72, 1.01)	**0.75 (0.60**, **0.95)**	**0.70 (0.54**, **0.92)**	**30 (8**, **46)**
Primary care consultation for COPD exacerbation	No	275.20 (244.47, 309.80)	1.00	1.00	1.00	–
	Yes	271.62 (255.84, 288.37)	0.98 (0.83, 1.15)	0.89 (0.74, 1.06)	0.87 (0.72, 1.05)	13 (−5, 28)
Hospitalization for pneumonia	No	17.51 (15.46, 19.82)	1.00	1.00	1.00	–
	Yes	22.15 (20.41, 24.04)	1.25 (1.07, 1.46)	**0.54 (0.45**, **0.64)**	**0.61 (0.50**, **0.75)**	**39 (25**, **50)**
Hospitalization for COPD exacerbation	No	122.17 (96.97, 153.91)	1.00	1.00	1.00	**–**
	Yes	98.52 (86.82, 111.81)	0.74 (0.53, 1.03)	**0.58 (0.42**, **0.82)**	**0.67 (0.46**, **0.99)**	**33 (1**, **54)**
All-cause death	No	21.82 (20.06, 23.74)	1.00	1.00	1.00	**–**
	Yes	22.23 (20.91, 23.64)	1.02 (0.91, 1.13)	**0.47 (0.41**, **0.54)**	**0.56 (0.48**, **0.65)**	**44 (35**, **52)**
Deaths due to pneumonia	No	6.44 (5.13, 7.90)	1.00	1.00	1.00	**–**
	Yes	6 (5.13, 7.02)	0.94 (0.72, 1.22)	**0.37 (0.27**, **0.51)**	**0.48 (0.33**, **0.71)**	**52 (29**, **67)**

AIRD: autoimmune rheumatic disease; COPD: chronic obstructive pulmonary disease; HR: hazard ratio; ILI: influenza-like illness; LRTI: lower respiratory tract infection. Significant results are in bold

a Model1: Adjusted for propensity score for IIV and year.

b Model2: Adjusted for propensity score for IIV and year with PS trimmed tails.

c Model3: VE: Vaccine effectiveness from Model 2.

### PS-matched analysis

Charlson comorbidity index, diabetes, chronic heart and renal diseases had *d *>* *0.10 between vaccinated and unvaccinated influenza cycles, and were included as additional covariates. The results of PS-matched analyses were consistent with PS-adjusted analyses, with the exception of a lack of statistically significant protective effect on ILI during IAPs (Tables 4 and 5).

**Table 4:** Influenza vaccine effectiveness in AIRDs using data from entire influenza cycle: propensity score-matched analysis

Outcomes	Vaccinated	Event rate (95% CI)/1000 person-years	Model 1 HR (95% CI)^a^	Model 2 HR (95% CI)^b^	Adjusted VE % (95% CI)^b^
Primary care consultation for LRTI requiring antibiotics	No	78.36 (73.40, 83.66)	1.00	1.00	–
	Yes	92.41 (87.30, 97.82)	1.18 (1.08, 1.29)	1.11 (1.02, 1.22)	−11 (−2, −22)
Primary care consultation for ILI	No	8.41 (6.92, 10.22)	1.00	1.00	–
	Yes	8.05 (6.67, 9.71)	0.97 (0.74, 1.27)	0.91 (0.69, 1.22)	9 (−22, 31)
Primary care consultation for COPD exacerbation	No	268.07 (224.64, 319.88)	1.00	1.00	–
	Yes	275.42 (242.56, 312.73)	1.02 (0.82, 1.27)	1.03 (0.82, 1.29)	−3 (−29, 18)
Hospitalization for pneumonia	No	25.44 (21.91, 29.54)	1.00	1.00	**–**
	Yes	15.81 (13.21, 18.93)	**0.62 (0.49**, **0.78)**	**0.50 (0.39**, **0.63)**	**50 (37**, **61)**
Hospitalization for COPD exacerbation	No	154.37 (115.26, 206.75)	1.00	1.00	**–**
	Yes	78.80 (58.44, 106.26)	**0.50 (0.34**, **0.75)**	**0.46 (0.31**, **0.70)**	**54 (30**, **69)**
All-cause death	No	32.22 (29.17, 35.59)	1.00	1.00	**–**
	Yes	18.94 (16.76, 21.40)	**0.59 (0.50**, **0.69)**	**0.45 (0.38**, **0.53)**	**55 (47**, **62)**
Deaths due to pneumonia	No	9.10 (7.09, 11.67)	1.00	1.00	**–**
	Yes	4.88 (3.54, 6.74)	**0.54 (0.36**, **0.81)**	**0.43 (0.29**, **0.66)**	**57 (34**, **71)**

a Adjusted for year and including patient ID as a clustering term.

b As in Model 1, and additionally adjusted for age, Charlson comorbidity index, diabetes, chronic heart disease and chronic kidney disease. AIRD: autoimmune rheumatic disease; COPD: chronic obstructive pulmonary disease; HR: hazard ratio; ILI: influenza-like illness; LRTI: lower respiratory tract infection. Significant results are in bold.

**Table 5:** Influenza vaccine effectiveness in AIRDs restricted to influenza-active periods: propensity score-matched analysis

Outcomes	Vaccinated	Event rate (95% CI)/1000 person-years	Model 1 HR (95% CI)^a^	Model 2 HR (95% CI) ^b^	Adjusted VE % (95% CI)^b^
Primary care consultation for LRTI requiring antibiotics	No	89.45 (83.09, 96.31)	1	1	–
	Yes	101.81 (95.38, 108.69)	1.14 (1.03, 1.26)	1.08 (0.98, 1.20)	−8 (−20, 2)
Primary care consultation for ILI	No	10.61 (8.59, 13.11)	1	1	–
	Yes	8.67 (6.95, 10.80)	0.83 (0.61, 1.12)	0.79 (0.57, 1.08)	21 (−8, 43)
Primary care consultation for COPD exacerbation	No	283.66 (232.23, 346.47)	1	1	–
	Yes	273.01 (234.91, 317.30)	0.95 (0.74, 1.22)	0.97 (0.75, 1.25)	3 (−25, 25)
Hospitalization for pneumonia	No	27.95 (23.50, 33.23)	1		**–**
	Yes	18.14 (14.79, 22.26)	**0.64 (0.49**, **0.84)**	**0.52 (0.39**, **0.69)**	**48 (31**, **61)**
Hospitalization for COPD exacerbation	No	161.41 (114.75, 227.04)	1	1	–
	Yes	85.03 (60.13, 120.23)	**0.52 (0.32**, **0.82)**	**0.50 (0.30**, **0.78)**	**50 (22**, **70)**
All-cause death	No	36.25 (32.34, 40.63)	1	1	**–**
	Yes	18.80 (16.19, 21.83)	**0.52 (0.43**, **0.63)**	**0.41 (0.33**, **0.50)**	**59 (50**, **67)**
Deaths due to pneumonia	No	11.51 (9.00, 15.07)	1	**1**	**–**
	Yes	5.11 (3.48, 7.50)	**0.44 (0.28**, **0.71)**	**0.37 (0.23**, **0.59)**	**63 (41**, **77)**

a Adjusted for year and including patient ID as a clustering term.

b As in Model 1, and, additionally adjusted for age, Charlson comorbidity index, diabetes, chronic heart and chronic kidney disease. AIRD: autoimmune rheumatic disease; COPD: chronic obstructive pulmonary disease; HR: hazard ratio; ILI: influenza-like illness; LRTI: lower respiratory tract infection. Significant results are in bold.

There was negative association between vaccination and all-cause mortality in pre-IAP and post-IAP, and between vaccination and hospitalization for pneumonia and COPD exacerbations in post-IAP (Table 6).

**Table 6:** Influenza vaccine effectiveness in pre-IAP, IAP and post-IAP: propensity score-matched analysis

Outcomes	Adjusted HR (95% CI)^a^		
	Pre-IAP	IAP	Post-IAP
Primary care consultation for LRTI requiring antibiotics	1.24 (0.55, 2.81)	1.08 (0.98, 1.20)	1.19 (0.98, 1.44)
Primary care consultation for ILI	3.02 (0.32, 28.22)	0.79 (0.57, 1.08)	1.49 (0.71, 3.16)
Primary care consultation for COPD exacerbation	1.20 (0.29, 5)	0.97 (0.75, 1.25)	1.46 (0.89, 2.38)
Hospitalization for pneumonia	0.19 (0.02, 1.66)	0.52 (0.39, 0.69)	0.50 (0.30, 0.83)
Hospitalization for COPD exacerbation	–/–	0.50 (0.30, 0.78)	0.51 (0.25, 1.05)
All cause death	0.11 (0.02, 0.57)	0.41 (0.33, 0.50)	0.67 (0.48, 0.93)
Deaths due to pneumonia	–/–	0.37 (0.23, 0.59)	0.88 (0.33, 2.35)

a Adjusted for year, age, Charlson comorbidity index, diabetes, chronic heart disease and chronic kidney disease, and including patient ID as a clustering term (Model 2). –/–: no outcomes; COPD: chronic obstructive pulmonary disease; HR: hazard ratio; IAP: influenza active periods; ILI: influenza-like illness; LRTI: lower respiratory tract infection.

### Sensitivity analyses

IIV protected from hospitalization for pneumonia, COPD exacerbation, and all-cause and pneumonia-related mortality in people with RA; when influenza cycles exposed to SSZ alone were excluded, and in the over 65s (supplementary Tables S3–S5, available at *Rheumatology* online). IIV reduced the risk of hospitalization for pneumonia and death in influenza cycles preceded by CS prescriptions, while there was a trend for reduction in hospitalization for COPD exacerbations (supplementary Table S6, available at *Rheumatology* online). The associations remained unchanged on IPTW using PS (supplementary Table S7, available at *Rheumatology* online).

## Discussion

This study reports that IIV reduces the risk of ILI by 30%, hospitalization for pneumonia by 39%, hospitalization for COPD exacerbations by 33% and death due to pneumonia by 52% in immunosuppressed AIRD patients during IAPs. Similar results were observed when follow-up was extended to the entire influenza cycle, except for absence of protective effect on ILI. This observation provides validity to the findings as the protective effect on ILI is not expected to extend beyond IAP. The protective effect of IIV was present when the analysis was restricted to people with greater immune dysfunction, e.g. diagnosed with RA, exposed to CS, prescribed potent DMARDs and age >65 years. We also observed a protective effect of vaccination on all-cause mortality. However, this is likely to be due to residual confounding as IIV associated with significantly reduced all-cause mortality in the pre-IAP when protection is not expected. Similarly, a negative association between vaccination and hospitalization for pneumonia and COPD exacerbations in the post-IAP raises the possibility that residual confounding may be present for these outcomes as well. This residual confounding could be due to several reasons such as healthy user bias, and selective non-prescribing to people with poor functional status and short life-expectancy, e.g. due to terminal illness, or the hospitalized [30].

We performed both PS-adjusted, IPTW and PS-matched analyses. The findings of PS-matched analysis were consistent with PS-adjusted and IPTW analysis, except for a lack of protective effect on ILI in the IAPs. However, this may be related to a >70% reduction in sample-size on PS-matching.

This large population-based study provides data on the effectiveness of IIV in AIRDs. A previous study only included <5% AIRD cases [11], and a recent smaller study reported 35% VE for hospitalization due to septicaemia, bacteraemia or viremia, and 38% VE for all-cause mortality with IIV in people with RA [31].

Our estimate of VE against ILI is comparable to those observed in healthy adults [3, 5]. VE is lower when ILI, a non-specific outcome with considerable imprecision around diagnosis, is the outcome rather than laboratory-confirmed influenza. ILI includes infections due to influenza and other respiratory viruses, and, as the IIV only targets the influenza virus, VE is lower for ILI than for laboratory-confirmed influenza [3, 5]. For example, in an RCT, the VE for laboratory-confirmed influenza was 50% (1997/8) and 86% (1998/9), but VE for ILI was only 10 and 33%, respectively [32]. ILI cases presenting to GPs in the UK do not routinely undergo virological investigations and data on laboratory-confirmed influenza are not available. Observational studies report greater VE for complications of influenza such as pneumonia (27–45%) and death (38–48%) than for ILI [11–13]. Our estimates of VE for pneumonia and death are of similar magnitude.

Primary care consultation for LRTI was selected as an outcome because bacterial chest infection is a complication of influenza. However, there was no evidence for protective effect on this outcome. This was unexpected, as IIV associated negatively with hospitalization and death due to pneumonia. It is possible that risk-averse antibiotic prescription to immunosuppressed people in primary care contributed. Similarly, our finding of no evidence of VE in those aged <45 years could be due to very few events in this age group. However, protective effects of IIV in young adults have been demonstrated [5].

Our study demonstrates that IIV is more effective in AIRDs than in diabetes [33]. This may be due to the fact that people with diabetes are at a high risk of influenza and its complications due to underlying immune dysfunction [34, 35]. However, VE in our study was comparable to that in healthy adults >65 years in age [3, 13, 36], despite reports of reduced serological response to IIV in the elderly [37].

The humoral and T cell responses to IIV are maintained in AIRD patients treated with MTX or anti-TNFα agents; however, B cell depletion therapy results in maintained T cell responses but lower humoral responses, and reduces the serological response to vaccination [16, 38, 39]. A recent RCT demonstrated greater antibody titres when MTX was temporarily discontinued post-vaccination [40]. However, 74 and 100% of patients in the intervention arm developed protective titres to the H1N1 and H3N2 viruses [40]. Prior to this study, there was no evidence that discontinuing MTX would boost serological response to IIV, and patients in the UK continue MTX post-vaccination. Thus, our findings together with the results of previous studies suggest that the serological response in immunosuppressed AIRD patients appears sufficient to offer protection from influenza and its complications.

In this study, 69.8% of influenza cycles were in receipt of an IIV. This may be due to financial incentives provided by the government to GPs under the Quality and Outcomes Framework to vaccinate people with comorbidities, e.g. diabetes, asthma or the elderly. AIRDs are not included in this list of comorbidities, although GPs retain discretion on whom to vaccinate. Consequently, only half of immunosuppressed people with AIRDs younger than 65 years old receive the IIV, often quite late in the course of the flu season [23].

The effectiveness of IIV varies according to the circulating influenza strain [4]. Although we could not assess VE by influenza strain, our results are encouraging. The findings of this study, together with the results of our previous study demonstrating the safety of IIV in people with AIRDs, provides evidence to promote seasonal flu vaccination in this population [41].

Ideally, vaccine efficacy should be assessed in RCTs, and the challenges in assessing VE using observational data are well known. Nevertheless, there are several strengths of this study, which include a large nationally representative sample, use of combination of diagnostic and prescription codes, and inclusion of broad-spectrum of AIRDs. Studies of VE are biased due to confounding by indication and healthy user bias, but we attempted to account for this using PS for vaccination, and the results of IAP-restricted analysis suggest that our findings are confounded for all-cause mortality, and potentially also for hospitalization for pneumonia and COPD exacerbation for which there was evidence of a protective effect in the post-IAP. In this study, analyses were undertaken in data from all nine influenza cycles included in a single dataset. This *a priori* approach gave a more powerful analysis and increased precision for less common outcomes. Sensitivity and subgroup analyses confirmed protective effects in presence of greater immunosuppression. Finally, the results were consistent across PS-adjusted, IPTW using PS, and PS-matched analyses, providing internal validity.

However, this study has several limitations. We merged data from multiple influenza cycles and provide a single VE estimate for each outcome. However, we accounted for within-person correlations, and report robust standard errors. Penicillamine and gold were excluded from the DMARD list, however they are rarely prescribed nowadays. Vaccinations occurring outside the GP surgery may not be recorded in the CPRD, resulting in misclassification of vaccinated cycles as unvaccinated. However, this biases the results towards null rather than inflating VE estimates given our findings. Additionally, as data on prescription of biological agents are not recorded in the CPRD, we were unable to assess their impact on VE. However, a proportion of people included in the study are expected to be treated with biologics. Similarly, exclusion of the 2009–10 flu season implies that our results cannot be generalized to pandemic influenza. Although we controlled for confounding, unmeasured confounding and healthy user bias could have inflated VE [30]. Finally, VE estimates from observational studies do not equate to vaccine efficacy at population level. This is due to covariate imbalance in PS-adjusted analysis that cannot be entirely accounted for by covariate adjustment, and the fact that PS-matched analysis is restricted to a sample that differs from the entire population. However, it is ethically challenging to justify an RCT of vaccination in this at-risk population.

In conclusion, IIV prevents respiratory morbidity and mortality in immunosuppressed AIRD patients. Although the VE estimates reported here may be overestimated for hospitalization and mortality outcomes given the observational study design, people with AIRDs should be informed of the benefits of vaccination and offered IIV annually.

## Supplementary data

Supplementary data are available at *Rheumatology* online.

## Supplementary Material

keaa078_Supplementary_Data
