# Supplementary material for: Effectiveness of inactivated influenza vaccine in autoimmune rheumatic diseases treated with disease-modifying anti-rheumatic drugs
Source: Rheumatology (Oxford). 2020 Mar 11;59(12):3666–75. doi: 10.1093/rheumatology/keaa078 (PMC7733714; doi:10.1093/rheumatology/keaa078)
Supplement: keaa078_Supplementary_Data [file keaa078_supplementary_data.zip › keaa078-suppl_data/rhe-19-2088-File002.docx]

**SUPPLEMENTARY MATERIAL**

**Methods**

The Standardised difference (d) were calculated using the following formulae as described in “Austin PC. An Introduction to Propensity Score Methods for Reducing the Effects of Confounding in Observational Studies. Multivariate Behav Res. 2011;46(3):399–424.”

for proportions

$$d=\frac{p\text{treatment}-p\text{control}}{\sqrt[2]{(p\text{treatment}(1-p\text{treatment)}+p\text{control}(1-p\text{control)})\div2}}$$

for means

$$d=\frac{x\text{treatment}-x\text{control}}{\sqrt[2]{(({s\text{treatment}}^{2}+{s\text{control}}^{2})\div2}}$$

# **Supplementary Table S1: List of Read codes and medical codes used in this study.**

**Influenza vaccination**

*Read codes*

| **Medcode** | **Readcode** | **Readterm** |
| --- | --- | --- |
| 6 | 65E..00 | Influenza vaccination |
| 9039 | 9OX..00 | Influenza vacc. administratn. |
| 12104 | 9OX..11 | Flu vaccination administration |
| 12336 | ZV04800 | [V]Influenza vaccination |
| 21123 | ZV04811 | [V]Flu - influenza vaccination |
| 104688 | 65ED.00 | Seasonal influenza vaccination |
| 106994 | 65EE000 | Administration of first intranasal influenza vaccination |
| 106995 | 65EE100 | Administration of second intranasal influenza vaccination |
| 107156 | 65EE.00 | Administration of intranasal influenza vaccination |
| 107723 | 9N4q100 | DNA first intranasal seasonal influenza vaccination |

*Product codes*

| **Prodcode** | **Description** |
| --- | --- |
| 398 | Influenza inactivated split virion Vaccination (Aventis Pasteur MSD) |
| 639 | Influenza vaccine (split virion, inactivated) suspension for injection 0.5ml pre-filled syringes |
| 922 | Influenza inactivated surface antigen Vaccination |
| 1329 | Fluvirin vaccine suspension for injection 0.5ml pre-filled syringes (Novartis Vaccines and Diagnostics Ltd) |
| 2139 | Fluarix vaccine suspension for injection 0.5ml pre-filled syringes (GlaxoSmithKline UK Ltd) |
| 2552 | Influvac Sub-unit vaccine suspension for injection 0.5ml pre-filled syringes (Abbott Healthcare Products Ltd) |
| 2601 | Mfv-ject Vaccination (Aventis Pasteur MSD) |
| 9710 | Agrippal vaccine suspension for injection 0.5ml pre-filled syringes (Novartis Vaccines and Diagnostics Ltd) |
| 10030 | Inflexal V vaccine suspension for injection 0.5ml pre-filled syringes (Janssen-Cilag Ltd) |
| 11824 | Enzira vaccine suspension for injection 0.5ml pre-filled syringes (Pfizer Ltd) |
| 13595 | Fluzone Vaccination (Aventis Pasteur MSD) |
| 16585 | Viroflu vaccine suspension for injection 0.5ml pre-filled syringes (Janssen-Cilag Ltd) |
| 18612 | Mastaflu vaccine suspension for injection 0.5ml pre-filled syringes (Masta Ltd) |
| 24779 | Influenza inactivated split virion Paediatric vaccination |
| 27407 | Imuvac vaccine suspension for injection 0.5ml pre-filled syringes (Abbott Healthcare Products Ltd) |
| 30156 | Invivac vaccine suspension for injection 0.5ml pre-filled syringes (Abbott Healthcare Products Ltd) |
| 30198 | Influenza inactivated split virion Vaccination (sanofi pasteur MSD Ltd) |
| 32391 | Influenza vaccine (surface antigen, inactivated) suspension for injection 0.5ml pre-filled syringes (Novartis Vaccines and Diagnostics Ltd) |
| 38421 | Influenza inactivated split virion Vaccination (Evans Vaccines Ltd) |
| 40760 | Influenza vaccine (split virion, inactivated) 15microgram strain suspension for injection 0.1ml pre-filled syringes |
| 40876 | Influenza vaccine (split virion, inactivated) 9microgram strain suspension for injection 0.1ml pre-filled syringes |
| 41168 | Influenza H1N1 vaccine (split virion, inactivated, adjuvanted) emulsion and suspension for emulsion for injection |
| 41240 | Influenza H1N1 vaccine (whole virion, Vero cell derived, inactivated) suspension for injection |
| 43825 | Intanza 15microgram strain vaccine suspension for injection 0.1ml pre-filled syringes (sanofi pasteur MSD Ltd) |
| 43827 | Intanza 9microgram strain vaccine suspension for injection 0.1ml pre-filled syringes (sanofi pasteur MSD Ltd) |
| 44759 | INFLUENZA PRE-FILLED SYRINGE |
| 45661 | Influenza vaccine (split virion, inactivated) suspension for injection 0.5ml pre-filled syringes (Pfizer Ltd) |
| 47932 | Fluenz vaccine nasal suspension 0.2ml unit dose (AstraZeneca UK Ltd) |
| 48085 | Influenza inactivated split virion Vaccination (Chiron UK Ltd) |
| 48658 | Influenza vaccine (split virion, inactivated) suspension for injection 0.5ml pre-filled syringes (sanofi pasteur MSD Ltd) |
| 48740 | Influenza vaccine (surface antigen, inactivated) suspension for injection 0.5ml pre-filled syringes |
| 49716 | Influenza vaccine (surface antigen, inactivated, virosome) suspension for injection 0.5ml pre-filled syringes |
| 51087 | Optaflu vaccine suspension for injection 0.5ml pre-filled syringes (Novartis Vaccines and Diagnostics Ltd) |
| 51289 | Influenza vaccine (live attenuated) nasal suspension 0.2ml unit dose |
| 54677 | Preflucel vaccine suspension for injection 0.5ml pre-filled syringes (Baxter Healthcare Ltd) |
| 57140 | Influenza vaccine (live attenuated) nasal suspension 0.2ml unit dose |
| 57401 | Influvac Desu vaccine suspension for injection 0.5ml pre-filled syringes (Abbott Healthcare Products Ltd) |
| 57678 | Fluenz vaccine nasal suspension 0.2ml unit dose (AstraZeneca UK Ltd) |
| 57917 | Fluarix Tetra vaccine suspension for injection 0.5ml pre-filled syringes (GlaxoSmithKline UK Ltd) |
| 61580 | Influenza vaccine (split virion, inactivated) suspension for injection 0.25ml pre-filled syringes |
| 61792 | Fluenz Tetra vaccine nasal suspension 0.2ml unit dose (AstraZeneca UK Ltd) |
| 61898 | Influenza vaccine (split virion, inactivated) suspension for injection 0.5ml pre-filled syringes (A A H Pharmaceuticals Ltd) |
| 63690 | Inflexal V suspension for injection 0.5ml pre-filled syringes (sanofi pasteur MSD Ltd) |
| 65205 | FluMist Quadrivalent vaccine nasal suspension 0.2ml unit dose (AstraZeneca UK Ltd) |

**Influenza-like illness**

| **Medcode** | **Readcode** | **Readterm** |
| --- | --- | --- |
| 556 | H27..00 | Influenza |
| 2157 | H27z.11 | Flu like illness |
| 5947 | H27z.12 | Influenza like illness |
| 6094 | H2z..00 | Pneumonia or influenza NOS |
| 8980 | 16L..00 | Influenza-like symptoms |
| 10086 | H2...00 | Pneumonia and influenza |
| 11849 | H2y..00 | Other specified pneumonia or influenza |
| 13573 | H270000 | Influenza with bronchopneumonia |
| 14791 | H27y100 | Influenza with gastrointestinal tract involvement |
| 15774 | H271000 | Influenza with laryngitis |
| 15912 | H270.00 | Influenza with pneumonia |
| 16388 | H27z.00 | Influenza NOS |
| 23488 | H271z00 | Influenza with respiratory manifestations NOS |
| 27072 | 43dF.00 | Influenza A antibody level |
| 27073 | 43dG.00 | Influenza B antibody level |
| 29457 | H270.11 | Chest infection - influenza with pneumonia |
| 29617 | H271100 | Influenza with pharyngitis |
| 31363 | H27yz00 | Influenza with other manifestations NOS |
| 35745 | H270z00 | Influenza with pneumonia NOS |
| 43625 | H271.00 | Influenza with other respiratory manifestation |
| 45892 | 43k2.00 | Influenza A antigen level |
| 46157 | H27y000 | Influenza with encephalopathy |
| 47472 | H27y.00 | Influenza with other manifestations |
| 48453 | 43k3.00 | Influenza B antigen level |
| 55646 | G520300 | Acute myocarditis - influenzal |
| 62632 | H270100 | Influenza with pneumonia, influenza virus identified |
| 69335 | F030800 | Encephalitis due to influenza-specific virus not identified |
| 71658 | F030A00 | Encephalitis due to influenza-virus identified |
| 92944 | 4JDb.00 | Influenza (A&B) serology |
| 94930 | H29..00 | Avian influenza |
| 96017 | 4JU5.00 | Influenza B virus detected |
| 96018 | 4JU2.00 | Influenza H3 virus detected |
| 96019 | 4JU0.00 | Influenza H1 virus detected |
| 96599 | 4JU8.00 | Amantadine resistant virus detected |
| 97062 | 4JU4.00 | Influenza A virus, other or untyped strain detected |
| 97279 | Hyu0700 | [X]Influenza+other manifestations, virus not identified |
| 97605 | Hyu0600 | [X]Influenza+oth respiratory manifestatns,virus not identified |
| 97936 | Hyu0500 | [X]Influenza+other manifestations,influenza virus identified |
| 98102 | H2A..11 | Influenza A (H1N1) swine flu |
| 98103 | 1W0..00 | Possible influenza A virus H1N1 subtype |
| 98125 | 1J72.00 | Suspected influenza A virus subtype H1N1 infection |
| 98129 | H2A..00 | Influenza due to Influenza A virus subtype H1N1 |
| 98143 | 4J3L.00 | Influenza A virus H1N1 subtype detected |
| 98156 | 4JU3.00 | Influenza H5 virus detected |
| 98257 | Hyu0400 | [X]Flu+oth respiratory manifestations,'flu virus identified |
| 98331 | 4J3M.00 | Influenza A virus H1N1 subtype not detected |
| 99840 | 4JU6.00 | Oseltamivir resistant virus detected |
| 101845 | 4JDe.00 | Influenza A virus subtype H1N1 serology |
| 102918 | 4JU1.00 | Influenza H2 virus detected |
| 104016 | 43w6.00 | Influenza A nucleic acid detection |
| 105541 | 43wD.00 | Influenza B nucleic acid detection |

**Lower respiratory tract infections**

| **Medcode** | **Readcode** | **Readterm** |
| --- | --- | --- |
| 68 | H06z011 | Chest infection |
| 152 | H302.00 | Wheezy bronchitis |
| 293 | H06z111 | Respiratory tract infection |
| 312 | H060.00 | Acute bronchitis |
| 556 | H27..00 | Influenza |
| 1019 | H061.00 | Acute bronchiolitis |
| 1382 | H060w00 | Acute viral bronchitis unspecified |
| 2476 | H07..00 | Chest cold |
| 2581 | H06z000 | Chest infection NOS |
| 3163 | H300.00 | Tracheobronchitis NOS |
| 3358 | H06z100 | Lower resp tract infection |
| 4519 | 14B3.11 | H/O: bronchitis |
| 4899 | H06z200 | Recurrent chest infection |
| 5818 | 17...00 | Respiratory symptoms |
| 5909 | H312011 | Chronic wheezy bronchitis |
| 5978 | H060.11 | Acute wheezy bronchitis |
| 6124 | H062.00 | Acute lower respiratory tract infection |
| 6475 | R06..00 | [D]Respiratory system and chest symptoms |
| 7074 | H5yy.11 | Respiratory infection NOS |
| 7092 | H30..12 | Recurrent wheezy bronchitis |
| 8025 | H0...00 | Acute respiratory infections |
| 9043 | H060600 | Acute pneumococcal bronchitis |
| 11072 | H060300 | Acute purulent bronchitis |
| 14791 | H27y100 | Influenza with gastrointestinal tract involvement |
| 15626 | H310000 | Chronic catarrhal bronchitis |
| 15774 | H271000 | Influenza with laryngitis |
| 16388 | H27z.00 | Influenza NOS |
| 16468 | R06zz00 | [D]Respiratory system and chest symptoms NOS |
| 17185 | H061200 | Acute bronchiolitis with bronchospasm |
| 17359 | H30..11 | Chest infection - unspecified bronchitis |
| 17917 | H061z00 | Acute bronchiolitis NOS |
| 18451 | H061500 | Acute bronchiolitis due to respiratory syncytial virus |
| 20198 | H060z00 | Acute bronchitis NOS |
| 21061 | H3y0.00 | Chronic obstruct pulmonary dis with acute lower resp infectn |
| 21113 | H0z..00 | Acute respiratory infection NOS |
| 21145 | H060400 | Acute croupous bronchitis |
| 21492 | H060800 | Acute haemophilus influenzae bronchitis |
| 23488 | H271z00 | Influenza with respiratory manifestations NOS |
| 23663 | 17ZZ.00 | Respiratory symptom NOS |
| 24316 | H24..11 | Chest infection with infectious disease EC |
| 24800 | H060x00 | Acute bacterial bronchitis unspecified |
| 25603 | H310.00 | Simple chronic bronchitis |
| 27819 | H312.00 | Obstructive chronic bronchitis |
| 29273 | H060C00 | Acute bronchitis due to parainfluenza virus |
| 29617 | H271100 | Influenza with pharyngitis |
| 29669 | H06..00 | Acute bronchitis and bronchiolitis |
| 31363 | H27yz00 | Influenza with other manifestations NOS |
| 31886 | H060A00 | Acute bronchitis due to mycoplasma pneumoniae |
| 37447 | H06z112 | Acute lower respiratory tract infection |
| 41137 | H06z.00 | Acute bronchitis or bronchiolitis NOS |
| 43362 | H060700 | Acute streptococcal bronchitis |
| 43625 | H271.00 | Influenza with other respiratory manifestation |
| 44525 | H312z00 | Obstructive chronic bronchitis NOS |
| 44611 | D410300 | Polycythaemia due to cyanotic respiratory disease |
| 46052 | H20y000 | Severe acute respiratory syndrome |
| 46157 | H27y000 | Influenza with encephalopathy |
| 46397 | P8z..00 | Respiratory system anomaly NOS |
| 47472 | H27y.00 | Influenza with other manifestations |
| 47638 | R06z.00 | [D]Other respiratory system and chest symptoms |
| 48593 | H060D00 | Acute bronchitis due to respiratory syncytial virus |
| 49794 | H060900 | Acute neisseria catarrhalis bronchitis |
| 54533 | H061000 | Acute capillary bronchiolitis |
| 54739 | Hyu8400 | [X]Respiratory disorders in other diseases CE |
| 61118 | H310z00 | Simple chronic bronchitis NOS |
| 63697 | 43jQ.00 | Avian influenza virus nucleic acid detection |
| 64716 | 7N22y00 | [SO]Specified respiratory tract NEC |
| 64890 | H060E00 | Acute bronchitis due to rhinovirus |
| 65916 | H060F00 | Acute bronchitis due to echovirus |
| 66043 | H31y.00 | Other chronic bronchitis |
| 66228 | H061600 | Acute bronchiolitis due to other specified organisms |
| 66397 | Hyu1.00 | [X]Other acute lower respiratory infections |
| 69192 | H061300 | Acute exudative bronchiolitis |
| 70630 | Hyu8.00 | [X]Other diseases of the respiratory system |
| 70815 | H464z00 | Chronic respiratory conditions due to chemical fumes NOS |
| 71370 | H060200 | Acute pseudomembranous bronchitis |
| 73100 | Hyu1000 | [X]Acute bronchitis due to other specified organisms |
| 91123 | 43jz.00 | Parainfluenza type 3 nucleic acid detection |
| 93153 | H060B00 | Acute bronchitis due to coxsackievirus |
| 94130 | 43jx.00 | Parainfluenza type 1 nucleic acid detection |
| 94858 | 43jy.00 | Parainfluenza type 2 nucleic acid detection |
| 94930 | H29..00 | Avian influenza |
| 96017 | 4JU5.00 | Influenza B virus detected |
| 96018 | 4JU2.00 | Influenza H3 virus detected |
| 96019 | 4JU0.00 | Influenza H1 virus detected |
| 96286 | 4JUF.00 | Human parainfluenza virus detected |
| 97062 | 4JU4.00 | Influenza A virus, other or untyped strain detected |
| 97279 | Hyu0700 | [X]Influenza+other manifestations, virus not identified |
| 97605 | Hyu0600 | [X]Influenza+oth respiratory manifestatns,virus not identifd |
| 97936 | Hyu0500 | [X]Influenza+other manifestations,influenza virus identified |
| 98102 | H2A..11 | Influenza A (H1N1) swine flu |
| 98103 | 1W0..00 | Possible influenza A virus H1N1 subtype |
| 98115 | 1J72.11 | Suspected swine influenza |
| 98125 | 1J72.00 | Suspected influenza A virus subtype H1N1 infection |
| 98129 | H2A..00 | Influenza due to Influenza A virus subtype H1N1 |
| 98143 | 4J3L.00 | Influenza A virus H1N1 subtype detected |
| 98156 | 4JU3.00 | Influenza H5 virus detected |
| 98257 | Hyu0400 | [X]Flu+oth respiratory manifestations,'flu virus identified |
| 99214 | Hyu1100 | [X]Acute bronchiolitis due to other specified organisms |
| 99762 | 14B9.00 | History of acute lower respiratory tract infection |
| 101775 | H060100 | Acute membranous bronchitis |
| 103785 | H58y500 | Respiratory bronchiolitis associated interstitial lung dis |
| 572 | H26..00 | Pneumonia due to unspecified organism |
| 886 | H25..00 | Bronchopneumonia due to unspecified organism |
| 1576 | H231.00 | Pneumonia due to mycoplasma pneumoniae |
| 1849 | H21..00 | Lobar (pneumococcal) pneumonia |
| 3683 | H261.00 | Basal pneumonia due to unspecified organism |
| 4910 | H56y100 | Interstitial pneumonia |
| 5202 | H20..00 | Viral pneumonia |
| 5324 | H28..00 | Atypical pneumonia |
| 5612 | H224.00 | Pneumonia due to staphylococcus |
| 6094 | H2z..00 | Pneumonia or influenza NOS |
| 6181 | H061400 | Obliterating fibrous bronchiolitis |
| 9389 | H20..11 | Chest infection - viral pneumonia |
| 9639 | H260.00 | Lobar pneumonia due to unspecified organism |
| 10086 | H2...00 | Pneumonia and influenza |
| 11202 | H530z00 | Abscess of lung NOS |
| 11849 | H2y..00 | Other specified pneumonia or influenza |
| 12061 | H22y200 | Pneumonia - Legionella |
| 12423 | H223.00 | Pneumonia due to streptococcus |
| 13573 | H270000 | Influenza with bronchopneumonia |
| 14976 | H20z.00 | Viral pneumonia NOS |
| 15308 | A3A4.00 | Legionella |
| 15912 | H270.00 | Influenza with pneumonia |
| 16287 | H25..11 | Chest infection - unspecified bronchopneumonia |
| 17025 | H233.00 | Chlamydial pneumonia |
| 19400 | H26..11 | Chest infection - pnemonia due to unspecified organism |
| 19992 | AC21.00 | Lung echinococcus granulosus |
| 21185 | H53..00 | Abscess of lung and mediastinum |
| 22795 | H22..11 | Chest infection - other bacterial pneumonia |
| 23095 | H22z.00 | Bacterial pneumonia NOS |
| 23546 | H220.00 | Pneumonia due to klebsiella pneumoniae |
| 23726 | H24y700 | Pneumonia with varicella |
| 25694 | H23..00 | Pneumonia due to other specified organisms |
| 26125 | H312300 | Bronchiolitis obliterans |
| 27519 | H24y200 | Pneumonia with pneumocystis carinii |
| 27641 | A789300 | HIV disease resulting in Pneumocystis carinii pneumonia |
| 28634 | H22..00 | Other bacterial pneumonia |
| 29005 | H530.00 | Abscess of lung |
| 29166 | H21..11 | Chest infection - pneumococcal pneumonia |
| 29457 | H270.11 | Chest infection - influenza with pneumonia |
| 30437 | H243.00 | Pneumonia with whooping cough |
| 30591 | H221.00 | Pneumonia due to pseudomonas |
| 30653 | H23..11 | Chest infection - pneumonia organism OS |
| 31269 | H201.00 | Pneumonia due to respiratory syncytial virus |
| 32172 | A551.00 | Postmeasles pneumonia |
| 33478 | H20y.00 | Viral pneumonia NEC |
| 33730 | H530000 | Single lung abscess |
| 34251 | H23z.00 | Pneumonia due to specified organism NOS |
| 34274 | H246.00 | Pneumonia with aspergillosis |
| 34659 | H53z.00 | Abscess of lung and mediastinum NOS |
| 34732 | A054.00 | Amoebic lung abscess |
| 35082 | H243.11 | Pneumonia with pertussis |
| 35189 | H530300 | Abscess of lung with pneumonia |
| 35220 | AD63.00 | Pneumocystosis |
| 35745 | H270z00 | Influenza with pneumonia NOS |
| 36675 | H202.00 | Pneumonia due to parainfluenza virus |
| 37711 | H530100 | Multiple lung abscess |
| 37881 | H222.00 | Pneumonia due to haemophilus influenzae |
| 40299 | AB24.11 | Pneumonia - candidal |
| 40498 | H24..00 | Pneumonia with infectious diseases EC |
| 41034 | H240.00 | Pneumonia with measles |
| 41084 | A221.11 | Woolsorters' disease |
| 41404 | AB50100 | Primary pulmonary blastomycosis |
| 41589 | H061100 | Acute obliterating bronchiolitis |
| 43286 | H241.00 | Pneumonia with cytomegalic inclusion disease |
| 43884 | H22yz00 | Pneumonia due to bacteria NOS |
| 45161 | A221.00 | Pulmonary anthrax |
| 45425 | H22y100 | Pneumonia due to proteus |
| 47295 | A205.00 | Pneumonic plague, unspecified |
| 47973 | A54x400 | Herpes simplex pneumonia |
| 48481 | AB24.00 | Candidiasis of lung |
| 48804 | H222.11 | Pneumonia due to haemophilus influenzae |
| 49398 | H24y600 | Pneumonia with typhoid fever |
| 50408 | A730.00 | Ornithosis with pneumonia |
| 50867 | H22y.00 | Pneumonia due to other specified bacteria |
| 52071 | H247000 | Pneumonia with candidiasis |
| 52384 | H22yX00 | Pneumonia due to other aerobic gram-negative bacteria |
| 52520 | Hyu0800 | [X]Other viral pneumonia |
| 53753 | Hyu0H00 | [X]Other pneumonia, organism unspecified |
| 53947 | Hyu0D00 | [X]Pneumonia in viral diseases classified elsewhere |
| 53969 | H247z00 | Pneumonia with systemic mycosis NOS |
| 54540 | AB30.00 | Primary pulmonary coccidioidomycosis |
| 54551 | AB40700 | Chronic pulmonary histoplasmosis capsulati |
| 54906 | AB65000 | Pulmonary cryptococcosis |
| 57667 | H530200 | Gangrenous pneumonia |
| 58896 | A022200 | Salmonella pneumonia |
| 59951 | AB42.00 | Pulmonary histoplasmosis |
| 60119 | H230.00 | Pneumonia due to Eaton's agent |
| 60299 | H22y011 | E.coli pneumonia |
| 60482 | H24y300 | Pneumonia with Q-fever |
| 61623 | H24y000 | Pneumonia with actinomycosis |
| 62408 | AC12.11 | Lung fluke disease |
| 62623 | H242.00 | Pneumonia with ornithosis |
| 62632 | H270100 | Influenza with pneumonia, influenza virus identified |
| 63763 | Hyu0A00 | [X]Other bacterial pneumonia |
| 63858 | H223000 | Pneumonia due to streptococcus, group B |
| 64306 | A391.00 | Pulmonary actinomycosis |
| 65419 | H22y000 | Pneumonia due to escherichia coli |
| 66362 | H24z.00 | Pneumonia with infectious diseases EC NOS |
| 67836 | H200.00 | Pneumonia due to adenovirus |
| 67901 | H24y100 | Pneumonia with nocardiasis |
| 69782 | H24y.00 | Pneumonia with other infectious diseases EC |
| 70559 | H24yz00 | Pneumonia with other infectious diseases EC NOS |
| 70710 | A203.00 | Primary pneumonic plague |
| 72182 | H24y400 | Pneumonia with salmonellosis |
| 73340 | A39y000 | Pulmonary nocardiosis |
| 73735 | H232.00 | Pneumonia due to pleuropneumonia like organisms |
| 91481 | AB40600 | Acute pulmonary histoplasmosis capsulati |
| 96332 | AyuEU00 | [X]Other pulmonary aspergillosis |
| 98381 | Hyu0B00 | [X]Pneumonia due to other specified infectious organisms |
| 98782 | H24y500 | Pneumonia with toxoplasmosis |
| 100742 | AB63300 | Allergic bronchopulmonary aspergillosis |
| 101292 | AB41500 | Histoplasma duboisii with pneumonia |
| 101507 | AB40500 | Histoplasma capsulatum with pneumonia |
| 102918 | 4JU1.00 | Influenza H2 virus detected |
| 103404 | H247100 | Pneumonia with coccidioidomycosis |
| 104121 | H2B..00 | Community acquired pneumonia |
| 104264 | H2C..00 | Hospital acquired pneumonia |
| 106300 | H203.00 | Pneumonia due to human metapneumovirus |
| 106908 | H244.00 | Pneumonia with tularaemia |
| 111027 | Hyu0C00 | [X]Pneumonia in bacterial diseases classified elsewhere |
| 111655 | Hyu0G00 | [X]Pneumonia in other diseases classified elsewhere |

**Chronic obstructive pulmonary disease exacerbation**

| **Medcode** | **Readcode** | **Readterm** |
| --- | --- | --- |
| 1446 | H312200 | Acute exacerbation of chronic obstructive airways disease |
| 7884 | H3y1.00 | Chron obstruct pulmonary dis wth acute exacerbation, unspec |
| 11019 | 8H2R.00 | Admit COPD emergency |

**Chronic obstructive pulmonary disease**

| **Medcodes** | **Read Code** | **Read Term** |
| --- | --- | --- |
| 794 | H32..00 | Emphysema |
| 998 | H3...11 | Chronic obstructive airways disease |
| 1001 | H3...00 | Chronic obstructive pulmonary disease |
| 1446 | H312200 | Acute exacerbation of chronic obstructive airways disease |
| 3243 | H31..00 | Chronic bronchitis |
| 4084 | 663K.00 | Airways obstructn irreversible |
| 5710 | H3z..00 | Chronic obstructive airways disease NOS |
| 5798 | H312000 | Chronic asthmatic bronchitis |
| 5909 | H312011 | Chronic wheezy bronchitis |
| 7884 | H3y1.00 | Chron obstruct pulmonary dis wth acute exacerbation, unspec |
| 9177 | 663J.00 | Airways obstruction reversible |
| 9520 | 66YB.00 | Chronic obstructive pulmonary disease monitoring |
| 9876 | H38..00 | Severe chronic obstructive pulmonary disease |
| 10802 | H37..00 | Moderate chronic obstructive pulmonary disease |
| 10863 | H36..00 | Mild chronic obstructive pulmonary disease |
| 10980 | H322.00 | Centrilobular emphysema |
| 11150 | H311.00 | Mucopurulent chronic bronchitis |
| 11287 | 66YM.00 | Chronic obstructive pulmonary disease annual review |
| 12166 | H3y..00 | Other specified chronic obstructive airways disease |
| 14798 | H312100 | Emphysematous bronchitis |
| 15157 | H31z.00 | Chronic bronchitis NOS |
| 15626 | H310000 | Chronic catarrhal bronchitis |
| 16342 | 14B3.00 | H/O: chr.obstr. airway disease |
| 16410 | H32yz00 | Other emphysema NOS |
| 18476 | 66YL.11 | COPD follow-up |
| 18621 | 66YL.00 | Chronic obstructive pulmonary disease follow-up |
| 18792 | 9Oi..00 | Chronic obstructive pulmonary disease monitoring admin |
| 19434 | 1J71.00 | Suspected chronic obstructive pulmonary disease |
| 19721 | 8CE6.00 | Chronic obstructive pulmonary disease leaflet given |
| 21061 | H3y0.00 | Chronic obstruct pulmonary dis with acute lower resp infectn |
| 22905 | H581.00 | Interstitial emphysema |
| 23492 | H320z00 | Chronic bullous emphysema NOS |
| 24248 | H313.00 | Mixed simple and mucopurulent chronic bronchitis |
| 25603 | H310.00 | Simple chronic bronchitis |
| 26018 | 66YS.00 | Chronic obstructive pulmonary disease monitoring by nurse |
| 26306 | H320.00 | Chronic bullous emphysema |
| 27819 | H312.00 | Obstructive chronic bronchitis |
| 28755 | 9Oi0.00 | Chronic obstructive pulmonary disease monitoring 1st letter |
| 32727 | H33z.11 | Hyperreactive airways disease |
| 33450 | H32z.00 | Emphysema NOS |
| 34202 | 9Oi1.00 | Chronic obstructive pulmonary disease monitoring 2nd letter |
| 34215 | 9Oi2.00 | Chronic obstructive pulmonary disease monitoring 3rd letter |
| 37247 | H3z..11 | Chronic obstructive pulmonary disease NOS |
| 37371 | 66YD.00 | Chronic obstructive pulmonary disease monitoring due |
| 37959 | H311100 | Fetid chronic bronchitis |
| 38074 | 9Oi4.00 | Chronic obstructive pulmonary disease monitor phone invite |
| 40159 | H311000 | Purulent chronic bronchitis |
| 40788 | H32y.00 | Other emphysema |
| 42258 | 9Oi3.00 | Chronic obstructive pulmonary disease monitoring verb invite |
| 42313 | 679V.00 | Health education - chronic obstructive pulmonary disease |
| 44525 | H312z00 | Obstructive chronic bronchitis NOS |
| 45089 | H31y100 | Chronic tracheobronchitis |
| 45770 | 66Yg.00 | Chronic obstructive pulmonary disease disturbs sleep |
| 45771 | 66Yh.00 | Chronic obstructive pulmonary disease does not disturb sleep |
| 45777 | 8CR1.00 | Chronic obstructive pulmonary disease clini management plan |
| 45998 | 66YT.00 | Chronic obstructive pulmonary disease monitoring by doctor |
| 46248 | J650200 | Acute emphysematous cholecystitis |
| 46578 | H321.00 | Panlobular emphysema |
| 53851 | Hyu4200 | [X]Airway disease due to other specific organic dusts |
| 54893 | H582.00 | Compensatory emphysema |
| 56860 | H320000 | Segmental bullous emphysema |
| 59263 | H32y111 | Acute interstitial emphysema |
| 60188 | H320200 | Giant bullous emphysema |
| 61118 | H310z00 | Simple chronic bronchitis NOS |
| 61513 | H311z00 | Mucopurulent chronic bronchitis NOS |
| 63479 | H32y200 | MacLeod's unilateral emphysema |
| 64721 | H464000 | Chronic emphysema due to chemical fumes |
| 65733 | Hyu3100 | [X]Other specified chronic obstructive pulmonary disease |
| 66043 | H31y.00 | Other chronic bronchitis |
| 66058 | Hyu3000 | [X]Other emphysema |
| 67040 | H3y..11 | Other specified chronic obstructive pulmonary disease |
| 68066 | H31yz00 | Other chronic bronchitis NOS |
| 68662 | H320100 | Zonal bullous emphysema |
| 70787 | H32y100 | Atrophic (senile) emphysema |
| 92955 | H32y000 | Acute vesicular emphysema |
| 93568 | H39..00 | Very severe chronic obstructive pulmonary disease |
| 99536 | H320300 | Bullous emphysema with collapse |
| 101042 | 8BMW.00 | Issue of chronic obstructive pulmonary disease rescue pack |
| 102685 | 66YB000 | Chronic obstructive pulmonary disease 3 monthly review |
| 103007 | 66YB100 | Chronic obstructive pulmonary disease 6 monthly review |
| 103494 | 14B3.12 | History of chronic obstructive pulmonary disease |
| 103678 | 8BMa000 | Chronic obstructiv pulmonary disease medication optimisation |
| 104481 | 8CMV.00 | Has chronic obstructive pulmonary disease care plan |
| 104608 | H3A..00 | End stage chronic obstructive airways disease |
| 104710 | 9NgP.11 | On COPD (chr obstruc pulmonary disease) supportv cre pathway |
| 104985 | 9NgP.00 | On chronic obstructive pulmonary disease supprtv cre pathway |
| 104998 | 8I61000 | Chronic obstructve pulmonry disease rescue pack not indicatd |
| 105457 | 8CMW500 | Chronic obstructive pulmonary disease care pathway |
| 106637 | 9Nk7000 | Seen in chronic obstructive pulmonary disease clinic |
| 106945 | 8IEZ.00 | Chronic obstructive pulmonary disease rescue pack declined |
| 107877 | 8IEy.00 | Chronic obstructive pulmon dis wr self managem plan declined |
| 108586 | 66Yz100 | Chronic obstruct pulmonary disease management plan declined |
| 109774 | 66YB200 | Telehealth chronic obstructive pulmonary disease monitoring |
| 109958 | H3B..00 | Asthma-chronic obstructive pulmonary disease overlap syndrom |
| 110092 | K101400 | Emphysematous pyelonephritis |

**Corticosteroids**

| **Prodcode** | **Description** |
| --- | --- |
| 44 | Prednisolone 5mg gastro-resistant tablets |
| 95 | Prednisolone 5mg tablets |
| 186 | Dexamethasone 500micrograms/5ml oral solution |
| 557 | Prednisolone 2.5mg gastro-resistant tablets |
| 578 | Prednisolone 1mg tablets |
| 955 | Prednisolone 5mg soluble tablets |
| 1063 | Prednesol 5mg Tablet (Sovereign Medical Ltd) |
| 1280 | Dexamethasone 2mg tablets |
| 1971 | Betnesol 500microgram soluble tablets (Focus Pharmaceuticals Ltd) |
| 2044 | prednisone 2.5 mg tab |
| 2130 | Methylprednisolone 4mg tablets |
| 2368 | Prednisolone 2.5mg tablet |
| 2390 | prednisolone e/c 1 mg tab |
| 2704 | Prednisolone 25mg tablets |
| 2799 | prednisolone 10 mg tab |
| 2949 | Prednisone 5mg tablets |
| 3059 | prednisolone 50 mg tab |
| 3345 | Sintisone Tablet (Pharmacia Ltd) |
| 3557 | Prednisone 1mg tablets |
| 4779 | Dexamethasone 500microgram tablets |
| 4943 | Dexamethasone 2mg/5ml oral solution sugar free |
| 5157 | Dexamethasone 2mg/5ml oral solution |
| 5490 | Deltacortril 5mg gastro-resistant tablets (Alliance Pharmaceuticals Ltd) |
| 5913 | Deltacortril 2.5mg gastro-resistant tablets (Alliance Pharmaceuticals Ltd) |
| 7286 | Betamethasone 500microgram soluble tablets sugar free |
| 7584 | prednisolone 4 mg tab |
| 7710 | prednisolone 15 mg tab |
| 7934 | prednisone 30 mg tab |
| 8022 | PREDSOL 5 MG TAB |
| 8261 | Medrone 16mg tablets (Pfizer Ltd) |
| 9727 | Prednisolone 50mg tablets |
| 9994 | Decadron 500microgram tablets (Merck Sharp & Dohme Ltd) |
| 10552 | Methylprednisolone 16mg tablets |
| 10683 | Medrone 2mg tablets (Pfizer Ltd) |
| 10684 | Methylprednisolone 2mg tablets |
| 10864 | Betamethasone 500microgram tablets |
| 11149 | Betnelan 500microgram tablets (Focus Pharmaceuticals Ltd) |
| 13522 | prednisolone 2 mg tab |
| 13615 | prednisone 10 mg tab |
| 14172 | Methylprednisolone 100mg tablets |
| 15555 | Medrone 4mg tablets (Pfizer Ltd) |
| 16724 | prednisone 50 mg tab |
| 18042 | Medrone 100mg tablets (Pfizer Ltd) |
| 19141 | Prednisolone 5mg soluble tablets (AMCo) |
| 19562 | DEPO-MEDRONE (1ML) |
| 20095 | Precortisyl forte 25mg Tablet (Aventis Pharma) |
| 20670 | prednisolone e/c |
| 21218 | Dexsol 2mg/5ml oral solution (Rosemont Pharmaceuticals Ltd) |
| 21417 | Prednisolone 5mg tablets (A A H Pharmaceuticals Ltd) |
| 21833 | Decortisyl 5mg Tablet (Roussel Laboratories Ltd) |
| 21903 | Oradexon-organon 2mg Tablet (Organon Laboratories Ltd) |
| 23512 | Precortisyl 5mg Tablet (Hoechst Marion Roussel) |
| 24716 | prednisolone e/c |
| 25077 | DEPO-MEDRONE 40MG/ML (3ML) |
| 25272 | Precortisyl 1mg Tablet (Hoechst Marion Roussel) |
| 26691 | DEPO-MEDRONE (2ML) |
| 27889 | Prednisolone |
| 27959 | Prednisolone |
| 27962 | Deltastab 1mg Tablet (Waymade Healthcare Plc) |
| 28375 | Prednisolone 2.5mg gastro-resistant tablets (A A H Pharmaceuticals Ltd) |
| 28376 | Prednisolone 2.5mg Gastro-resistant tablet (Biorex Laboratories Ltd) |
| 28615 | METHYLPREDNISOLONE L/A 4 MG CAP |
| 28859 | Deltastab 5mg Tablet (Waymade Healthcare Plc) |
| 29333 | Prednisolone 5mg tablets (Actavis UK Ltd) |
| 30390 | deltastab 2 mg tab |
| 31327 | Prednisolone steaglate 6.65mg tablet |
| 31532 | Prednisolone 5mg gastro-resistant tablets (A A H Pharmaceuticals Ltd) |
| 32803 | Prednisolone 5mg gastro-resistant tablets (Actavis UK Ltd) |
| 32835 | Prednisolone 5mg tablets (Wockhardt UK Ltd) |
| 33691 | Prednisolone 5mg Gastro-resistant tablet (Biorex Laboratories Ltd) |
| 33988 | Prednisolone 5mg Tablet (Co-Pharma Ltd) |
| 33990 | Prednisolone 5mg Tablet (IVAX Pharmaceuticals UK Ltd) |
| 34109 | Prednisolone 5 mg gastro-resistant tablet |
| 34393 | Prednisolone 5mg gastro-resistant tablets (Teva UK Ltd) |
| 34404 | Prednisolone 1mg tablets (Actavis UK Ltd) |
| 34452 | Prednisolone 1mg tablets (A A H Pharmaceuticals Ltd) |
| 34461 | Prednisolone 2.5mg gastro-resistant tablets (Actavis UK Ltd) |
| 34631 | Prednisolone 1mg Tablet (Co-Pharma Ltd) |
| 34660 | Prednisolone 1mg tablets (Kent Pharmaceuticals Ltd) |
| 34748 | Prednisolone 1mg tablets (Teva UK Ltd) |
| 34781 | Prednisolone 5mg tablets (Kent Pharmaceuticals Ltd) |
| 34801 | Dexamethasone 0.5mg/5ml Oral solution (Rosemont Pharmaceuticals Ltd) |
| 34880 | Dexamethasone 2mg tablets (Aspen Pharma Trading Ltd) |
| 34914 | Prednisolone 1mg Tablet (Celltech Pharma Europe Ltd) |
| 34915 | Dexamethasone 500microgram tablets (Organon Laboratories Ltd) |
| 34978 | Prednisolone 1mg tablets (Wockhardt UK Ltd) |
| 36055 | Dexamethasone 2mg Tablet (Hillcross Pharmaceuticals Ltd) |
| 38407 | Prednisolone 20mg tablet |
| 41515 | Prednisolone 5mg tablets (Teva UK Ltd) |
| 41745 | Prednisolone 25mg tablets (Zentiva) |
| 43544 | Prednisone 5mg Tablet (Knoll Ltd) |
| 44380 | Prednisone 1mg modified-release tablets |
| 44723 | Prednisone 5mg modified-release tablets |
| 44802 | Lodotra 5mg modified-release tablets (Napp Pharmaceuticals Ltd) |
| 44803 | Lodotra 2mg modified-release tablets (Napp Pharmaceuticals Ltd) |
| 45234 | Dexamethasone 100microgram capsules |
| 45302 | Prednisolone 5mg Tablet (Biorex Laboratories Ltd) |
| 46711 | Prednisone 2mg modified-release tablets |
| 47142 | Prednisolone 5mg Soluble tablet (Amdipharm Plc) |
| 50225 | Betnesol 500microgram soluble tablets (Waymade Healthcare Plc) |
| 51753 | Prednisolone 1mg tablets (Strides Shasun (UK) Ltd) |
| 52396 | Dexamethasone 1mg/5ml oral solution |
| 53207 | Dexamethasone tablets |
| 53313 | Prednisolone 20mg/5ml oral suspension |
| 53336 | Prednisolone 25mg tablets (A A H Pharmaceuticals Ltd) |
| 54118 | Prednisolone 25mg/5ml oral suspension |
| 54432 | Lodotra 1mg modified-release tablets (Napp Pharmaceuticals Ltd) |
| 54434 | Prednisolone 2.5mg/5ml oral suspension |
| 54793 | Dexamethasone 2mg/5ml oral suspension |
| 55024 | Prednisolone 5mg/5ml oral solution |
| 55401 | Dexamethasone 500microgram tablets (A A H Pharmaceuticals Ltd) |
| 55480 | Prednisolone 2.5mg gastro-resistant tablets (Alliance Pharmaceuticals Ltd) |
| 56347 | Dexamethasone 5mg/5ml oral solution |
| 56443 | Dexamethasone 10mg/5ml oral solution |
| 56891 | Prednisolone 1mg tablets (Waymade Healthcare Plc) |
| 58000 | Prednisolone 5mg tablets (Almus Pharmaceuticals Ltd) |
| 58061 | Prednisone 50mg tablets |
| 58234 | Prednisolone 10mg/5ml oral solution |
| 58369 | Prednisolone 5mg tablets (Boston Healthcare Ltd) |
| 58384 | Prednisolone 1mg tablets (Almus Pharmaceuticals Ltd) |
| 58474 | Dexamethasone 2mg/5ml oral solution sugar free (A A H Pharmaceuticals Ltd) |
| 58987 | Prednisolone 5mg gastro-resistant tablets (Phoenix Healthcare Distribution Ltd) |
| 59229 | Dilacort 5mg gastro-resistant tablets (Auden McKenzie (Pharma Division) Ltd) |
| 59283 | Dilacort 2.5mg gastro-resistant tablets (Auden McKenzie (Pharma Division) Ltd) |
| 59338 | Prednisolone 1mg/5ml oral solution |
| 59912 | Prednisolone 5mg gastro-resistant tablets (Waymade Healthcare Plc) |
| 60064 | Dexamethasone 10mg/5ml oral solution sugar free |
| 60120 | Dexamethasone 2mg tablets (Alliance Healthcare (Distribution) Ltd) |
| 60421 | Prednisolone 5mg tablets (Strides Shasun (UK) Ltd) |
| 61132 | Prednisolone 1mg tablets (Boston Healthcare Ltd) |
| 61162 | Prednisolone 5mg tablets (Waymade Healthcare Plc) |
| 61689 | Prednisolone 5mg soluble tablets (A A H Pharmaceuticals Ltd) |
| 62656 | Prednisone 5mg Tablet (Hillcross Pharmaceuticals Ltd) |
| 62909 | Dexamethasone 2mg tablets (A A H Pharmaceuticals Ltd) |
| 63066 | Prednisolone 2.5mg tablets |
| 63214 | Prednisolone 5mg soluble tablets (Alliance Healthcare (Distribution) Ltd) |
| 63549 | Prednisolone 1mg/ml oral solution (Logixx Pharma Solutions Ltd) |
| 64007 | Pevanti 10mg tablets (AMCo) |
| 64008 | Pevanti 2.5mg tablets (AMCo) |
| 64050 | Martapan 2mg/5ml oral solution (Martindale Pharmaceuticals Ltd) |
| 64128 | Pevanti 5mg tablets (AMCo) |
| 64221 | Prednisolone 5mg/5ml oral suspension |
| 64235 | Betamethasone 500microgram soluble tablets sugar free (Alliance Healthcare (Distribution) Ltd) |
| 64747 | Dexamethasone 2mg/5ml oral solution |
| 64766 | Dexamethasone 20mg/5ml oral solution sugar free |
| 65626 | Prednisolone 10mg/5ml oral suspension |
| 66200 | Dexamethasone 2mg soluble tablets sugar free |
| 66287 | Dexamethasone 8mg soluble tablets sugar free |
| 66524 | Dexamethasone 4mg soluble tablets sugar free |
| 66550 | Prednisolone 5mg gastro-resistant tablets (Alliance Healthcare (Distribution) Ltd) |
| 66724 | Dexamethasone 10mg capsules |
| 67107 | Prednisolone 5mg gastro-resistant tablets (Alliance Pharmaceuticals Ltd) |
| 67559 | Prednisolone 5mg/5ml oral solution unit dose (A A H Pharmaceuticals Ltd) |
| 68103 | Dexamethasone 2mg soluble tablets sugar free (A A H Pharmaceuticals Ltd) |
| 68182 | Dexamethasone 2mg tablets (Teva UK Ltd) |
| 68306 | Betamethasone 500microgram soluble tablets sugar free (RPH Pharmaceuticals AB) |
| 68489 | Dexamethasone 4mg tablets |
| 68497 | Prednisolone 2.5mg gastro-resistant tablets (Waymade Healthcare Plc) |
| 68593 | Dexamethasone 5mg/5ml oral suspension |
| 68860 | Dexamethasone 8mg soluble tablets sugar free (A A H Pharmaceuticals Ltd) |
| 69572 | Dexamethasone 4mg/5ml oral suspension |
| 70611 | Dexamethasone 3mg/5ml oral solution |
| 70893 | Dexamethasone 40mg tablets |
| 71404 | Dexamethasone 2mg/5ml oral solution sugar free (Waymade Healthcare Plc) |
| 71926 | Dexamethasone 1.25mg/5ml oral suspension |
| 72537 | Dexamethasone 4mg/5ml oral solution |

# **Supplementary Table S2: Disease and demographic characteristics at study entry**

| **Patient characteristics** | **Number (%)**  **n=30, 788** |
| --- | --- |
| ***Sex*** |  |
| Female | 20,216 (65.66) |
| Male | 10,572 (34.34) |
| ***Age group (years)*** |  |
| <45 | 5,457 (17.72) |
| 45-64 | 13,969 (45.37) |
| >=65 | 11,362 (36.90) |
| ***Drugs*** |  |
| Methotrexate^1^ | 18,802 (61.07) |
| Sulfasalazine | 8,463 (27.49) |
| Leflunomide^1^ | 1,530 (4.97) |
| Azathioprine | 1,274 (4.14) |
| Others^2^ | 719 (2.34) |
| ***Rheumatic disease*** |  |
| RA | 23,242 (75.49) |
| Seronegative spondyloarthritis | 6,440 (20.92) |
| Lupus | 1,106 (3.59) |

# **Supplementary Table S3: Influenza vaccine effectiveness in Rheumatoid Arthritis using data from entire influenza-cycle**

| **Outcomes** | **Vaccinated** | **Events** | **Event rate (95% CI)/**  **1,000 person-years** | **Unadjusted HR**  **(95% CI)** | **Adjusted HR^1^**  **(95% CI)** | **Adjusted % VE^2^**  **(95% CI)** |  |
| --- | --- | --- | --- | --- | --- | --- | --- |
| Primary care consultation for LRTI requiring antibiotics | No | 3,019 | 83.88 (80.94 to 86.93) | 1.00 | 1.00 | - | |
|  | Yes | 5,357 | 104.74 (101.97 to 107.58) | 1.36 (1.28 to 1.44) | 1.05 (0.98 to 1.12) | -5 (-12 to 2) | |
| Primary care consultation for ILI | No | 258 | 6.98 (6.18 to 7.89) | 1.00 | 1.00 | - | |
|  | Yes | 355 | 6.53 (5.89 to 7.25) | 0.83(0.69 to 0.99) | **0.78 (0.61 to 1.00)** | **22 (0 to 39)** | |
| Primary care consultation for COPD exacerbation | No | 514 | 234.52 (215.10 to 255.70) | 1.00 | 1.00 | - | |
|  | Yes | 1,347 | 274 (260.25 to 289.58) | 1.16 (0.99 to 1.37) | 1.01 (0.85 to 1.18) | 1 (-18 to 15) | |
| Hospitalisation for pneumonia | No | 429 | 20.07 (18.25 to 22.06) | 1.00 | 1.00 | - | |
|  | Yes | 697 | 22.63 (21.01 to 24.38) | 1.21 (1.04 to 1.41) | **0.58 (0.49 to 0.68)** | **42 (32 to 51)** | |
| Hospitalisation for COPD exacerbation | No | 145 | 114.18 (97.03 to 134.36) | 1.00 | **1.00** | **-** | |
|  | Yes | 279 | 91.49 (81.36 to 102.89) | 0.78 (0.56 to 1.09) | **0.59 (0.42 to 0.82)** | **41 (18 to 58)** | |
| All cause death | No | 857 | 23.12 (21.62 to 24.72) | 1.00 | **1.00** | **-** | |
|  | Yes | 1,336 | 24.49 (23.21 to 25.84) | 0.98 (0.89 to 1.08) | **0.51 (0.45 to 0.58)** | **49 (42 to 55)** | |
| Deaths due to pneumonia | No | 145 | 1.85 (1.57 to 2.18) | 1.00 | **1.00** | **-** | |
|  | Yes | 201 | 1.77 (1.54 to 2.03) | 0.94 (0.74 to 1.20) | **0.46 (0.34 to 0.62)** | **54 (38 to 66)** | |

^1^Adjusted for propensity score for inactivated influenza vaccination and year, ^2^Vaccine effectiveness

# **Supplementary Table S4: Influenza vaccine effectiveness during entire influenza-cycle, excluding seasons with exposure to sulfasalazine alone**

| **Outcomes** | **Vaccinated** | **Events** | **Event rate (95% CI)/**  **1,000 person-years** | **Unadjusted HR**  **(95% CI)** | **Adjusted HR^1^**  **(95% CI)** | **Adjusted VE^2^**  **% (95% CI)** |
| --- | --- | --- | --- | --- | --- | --- |
| Primary care consultation for LRTI requiring antibiotics | No | 3,026 | 83.55 (80.62 to 86.58) | 1.00 | 1.00 | - |
|  | Yes | 5,390 | 102.33 (99.63 to 105.10) | 1.32 (1.24 to 1.40) | 1.06 (1.00 to 1.14) | -6 (-14 to 0) |
| Primary care consultation for ILI | No | 279 | 7.50 (6.67 to 8.43) | 1.00 | 1.00 | - |
|  | Yes | 410 | 7.34 (6.67 to 8.09) | 0.85 (0.72 to 1.01) | 0.86 (0.69 to 1.09) | 14 (-9 to 31) |
| Primary care consultation for COPD exacerbation | No | 423 | 223.59 (203.27 to 245.95) | 1.00 | 1.00 | - |
|  | Yes | 1125 | 259.27 (244.55 to 274.87) | 1.05 (0.88 to 1.26) | 0.99 (0.82 to 1.18) | 1 (-18 to 18) |
| Hospitalisation for pneumonia | No | 395 | 18.16 (16.46 to 20.04) | 1.00 | **1.00** | **-** |
|  | Yes | 607 | 19.14 (17.68 to 20.73) | 1.09 (0.93 to 1.27) | **0.55 (0.46 to 0.65)** | **45 (35 to 45)** |
| Hospitalisation for COPD exacerbation | No | 104 | 90.74 (74.88 to 109.97) | 1.00 | **1.00** | **-** |
|  | Yes | 214 | 78.10 (68.31 to 89.30) | 0.80 (0.55 to 1.17) | **0.57 (0.39 to 0.83)** | **43 (17 to 61)** |
| All cause death | No | 735 | 19.70 (18.32 to 21.17) | 1.00 | **1.00** | **-** |
|  | Yes | 1,114 | 19.86 (18.73 to 21.06) | 0.91 (0.82 to 1.01) | **0.49 (0.43 to 0.56)** | **51 (44 to 57)** |
| Deaths due to pneumonia | No | 126 | 5.77 (4.85 to 6.87) | 1.00 | **1.00** | **-** |
|  | Yes | 172 | 5.39 (4.64 to 6.25) | 0.91 (0.70 to 1.17) | **0.46 (0.33 to 0.63)** | **54 (37 to 67)** |

^1^Adjusted for propensity score for inactivated influenza vaccination and year, ^2^ Vaccine effectiveness

# **Supplementary Table S5: Influenza vaccine effectiveness using data from entire influenza-cycle and stratified according to age**

|  |  |  | **<45 years** |  |  | **45-64 years** |  |  | **≥65 years** |  |
| --- | --- | --- | --- | --- | --- | --- | --- | --- | --- | --- |
| **Outcomes** | **Vaccinated** | **Events**  **(rate/1,000 person-years)** | **Adjusted HR^1^**  **(95% CI)** | **Adjusted VE^2^**  **(95% CI)** | **Events**  **(rate/ 1,000 person-years)** | **Adjusted HR^1^**  **(95% CI)** | **Adjusted VE^2^**  **% (95% CI)** | **Events**  **(rate/ 1,000 person-years)** | **Adjusted HR^1^**  **(95% CI)** | **Adjusted VE^2^**  **(95% CI)** |
| Primary care consultation for LRTI requiring antibiotics | No | 449 (51.90) | 1.00 | - | 1,904 (77.52) | 1.00 | - | 1,470 (93.11) | 1.00 | - |
|  | Yes | 359 (71.01) | 1.24  (1.02 to 1.50) | -24  (-50 to -2) | 2,374 (96.05) | 1.01  (0.92 to 1.10) | -1  (-10 to 8) | 3,707 (108.99) | 1.09  (0.99 to 1.20) | -9  (-20 to 1) |
| Primary care consultation for ILI | No | 86 (9.76) | 1.00 | - | 178 (7.05) | 1.00 | - | 86 (5.31) | 1.00 | - |
|  | Yes | 62 (11.77) | 1.24  (0.81 to 1.92) | -24  (-92 to 19) | 199 (7.61) | 0.87  (0.65 to 1.16) | 13  (-16 to 35) | 218 (6.02) | 0.79  (0.53 to 1.17) | 21  (-17 to 47) |
| Primary care consultation for COPD exacerbation | No | 8 (175.64) | 1.00 | - | 211(251.07) | 1.00 | - | 388 (237.48) | 1.00 | - |
|  | Yes | 9 (204.73) | 0.93  (0.27 to 3.21) | 7  (-221 to 73) | 445 (284.75) | 0.97  (0.76 to 1.25) | 3  (-25 to 24) | 1102 (272.62) | 0.95  (0.79 to 1.15) | 5  (-15 to 21) |
| Hospitalised for pneumonia | No | 22 (4.37) | 1.00 | - | 109 (7.53) | 1.00 | - | 362 (38.36) | 1.00 | - |
|  | Yes | 8 (2.88) | 0.44  (0.16 to 1.21) | 56  (-21 to 84) | 108 (7.60) | **0.60**  **(0.43 to 0.85)** | **40**  **(15 to 57)** | 658 (31.33) | **0.51**  **(0.42 to 0.62)** | **49**  **(38 to 49)** |
| Hospitalisation for COPD exacerbation | No | 2(113.06) | * | * | 30 (60.77) | 1.00 | - | 131 (133.91) | 1.00 | - |
|  | Yes | 0 | * | * | 52(55.61) | **0.78**  **(0.44 to 1.38)** | 22  (-38 to 56) | 263 (101.12) | **0.59**  **(0.44 to 0.79)** | **41**  **(21 to 56)** |
| All cause death | No | 9 (1.02) | 1.00 | - | 137 (5.41) | 1.00 | - | 799 (49.27) | 1.00 | - |
|  | Yes | 5(0.94) | 1.14  (0.25 to 5.19) | -14  (-41 to 75) | 162 (6.17) | **0.67**  **(0.48 to 0.92)** | **33**  **(8 to 52)** | 1,311 (36.11) | **0.43**  **(0.37 to 0.49)** | **57**  **(51 to 63)** |
| Deaths due to pneumonia | No | 1 (0.20) | * | * | 19 (1.31) | 1.00 | - | 135 (14.21) | 1.00 | - |
|  | Yes | 0 | * | * | 14 (0.98) | 0.67  (0.28 to 1.58) | 33  (-58 to 72) | 206 (9.70) | **(0.27 to 0.53)** | **62**  **(47 to 73)** |

*Event numbers not sufficient to run any analysis. ^1^Adjusted for propensity score for inactivated influenza vaccination and year, ^2^Vaccine effectiveness

# **Supplementary Table S6**: Influenza vaccine effectiveness during influenza-cycles exposed to both DMARDs and oral corticosteroids

| **Outcomes** | **Vaccinated** | **Events** | **Event rate (95% CI)/ 1,000 person-years** | **HR (95% CI)** | **Adjusted HR^1^**  **(95% CI)** |
| --- | --- | --- | --- | --- | --- |
| Primary care consultation for LRTI requiring antibiotics | No | 516 | 113.69 (104.29 to 123.94) | 1.00 | 1.00 |
|  | Yes | 1,056 | 140.49 (132.27 to 149.23) | 1.28 (1.12 to 1.46) | 1.08 (0.92 to 1.26) |
| Primary care consultation for ILI | No | 43 | 9.16 (6.80 12.36) | 1.00 | 1.00 |
|  | Yes | 70 | 8.60 (6.80 to 10.87) | 0.75 (0.49 to 1.14) | 0.85 (0.50 to 1.42) |
| Primary care consultation for COPD exacerbation | No | 94 | 215.61 (176.14 to 263.91) | 1.00 | 1.00 |
|  | Yes | 252 | 235.87 (208.47 to 266.86) | 0.95 (0.67 to 1.34) | 0.84 (0.58 to 1.20) |
| Hospitalisation for pneumonia | No | 91 | 32.18 (26.20 to 39.52) | 1.00 | 1.00 |
|  | Yes | 166 | 34.68 (29.79 to 40.38) | 0.97 (0.72 to 1.32) | **0.61 (0.43 to 0.85)** |
| Hospitalisation for COPD exacerbation | No | 38 | 104.73 (197.81 to 81) | 1.00 | 1.00 |
|  | Yes | 86 | 123.53 (100 to 152.60) | 0.79 (0.39 to 1.59) | 0.64 (0.36 to 1.13) |
| All cause death | No | 125 | 26.53 (22.26 to 31.61) | 1.00 | 1.00 |
|  | Yes | 153 | 18.71 (15.96 to 21.92) | **0.57 (0.44 to 0.74**) | **0.44 (0.31 to 0.64)** |
| Deaths due to pneumonia | No | 27 | 9.46 (6.49 to 13.79) | 1.00 | 1.00 |
|  | Yes | 27 | 5.55 (3.80 to 8.09) | **0.46 (0.26 to 0.79)** | **0.23 (0.11 to 0.47)** |

^1^Adjusted for propensity score for inactivated influenza vaccination and year

# **SupplementaryTable S7: Influenza vaccine effectiveness: inverse probability treatment weighting using propensity score**

| **Outcomes** | **Vaccinated** | **Adjusted HR (95% CI)^1^** | |
| --- | --- | --- | --- |
|  |  | **Entire influenza-cycle** | **Influenza-active period** |
| Primary care consultation for LRTI requiring antibiotics | No | 1.00 | 1.00 |
|  | Yes | 1.13 (1.06 to 1.22) | 1.07 (0.98 to 1.16) |
| Primary care consultation for ILI | No | 1.00 | 1.00 |
|  | Yes | **0.78 (0.62 to 0.97)** | **0.71 (0.55 to 0.91)** |
| Primary care consultation for COPD exacerbation | No | 1.00 | 1.00 |
|  | Yes | 1.01 (0.84 to 1.23) | 0.86 (0.68 to 1.07) |
| Hospitalisation for pneumonia | No | 1.00 | 1.00 |
|  | Yes | **0.56 (0.47 to 0.67)** | **0.46 (0.38 to 0.57)** |
| Hospitalisation for COPD exacerbation | No | 1.00 | 1.00 |
|  | Yes | **0.53 (0.39 to 0.73)** | **0.49 (0.33 to 0.72)** |
| All cause death | No | 1.00 | 1.00 |
|  | Yes | **0.43 (0.39 to 0.49**) | **0.31 (0.27 to 0.35)** |
| Deaths due to pneumonia | No | 1.00 | 1.00 |
|  | Yes | **0.37 (0.27 to 0.49)** | **0.24 (0.17 to 0.33)** |

^1^Adjusted for year of vaccination and including participant ID as a clustering ter

# **Supplementary Table S8: Inactivated influenza vaccination patterns**

| No. of potential vaccinations missed | No (%) of participants  N=30,788 |
| --- | --- |
| 0 | 15,355 (49.87) |
| 1 | 6,989 (22.70) |
| 2 | 2,991 (9.71) |
| 3 | 2,064 (6.70) |
| 4 | 1,200 (3.90) |
| 5 | 768 (2.49) |
| 6 | 609 (1.98) |
| 7 | 344 (1.12) |
| 8 | 255 (0.83) |
| 9 | 213 (0.69) |

**Supplementary figure legend**

# **Supplementary Figure S1: Propensity for receiving influenza vaccination from 2006 to 2016 (excluding 2009/10 pandemic year)**
