# Supplementary figures and images for: Effectiveness of inactivated influenza vaccine in autoimmune rheumatic diseases treated with disease-modifying anti-rheumatic drugs
Source: Rheumatology (Oxford). 2020 Mar 11;59(12):3666–75. doi: 10.1093/rheumatology/keaa078 (PMC7733714; doi:10.1093/rheumatology/keaa078)

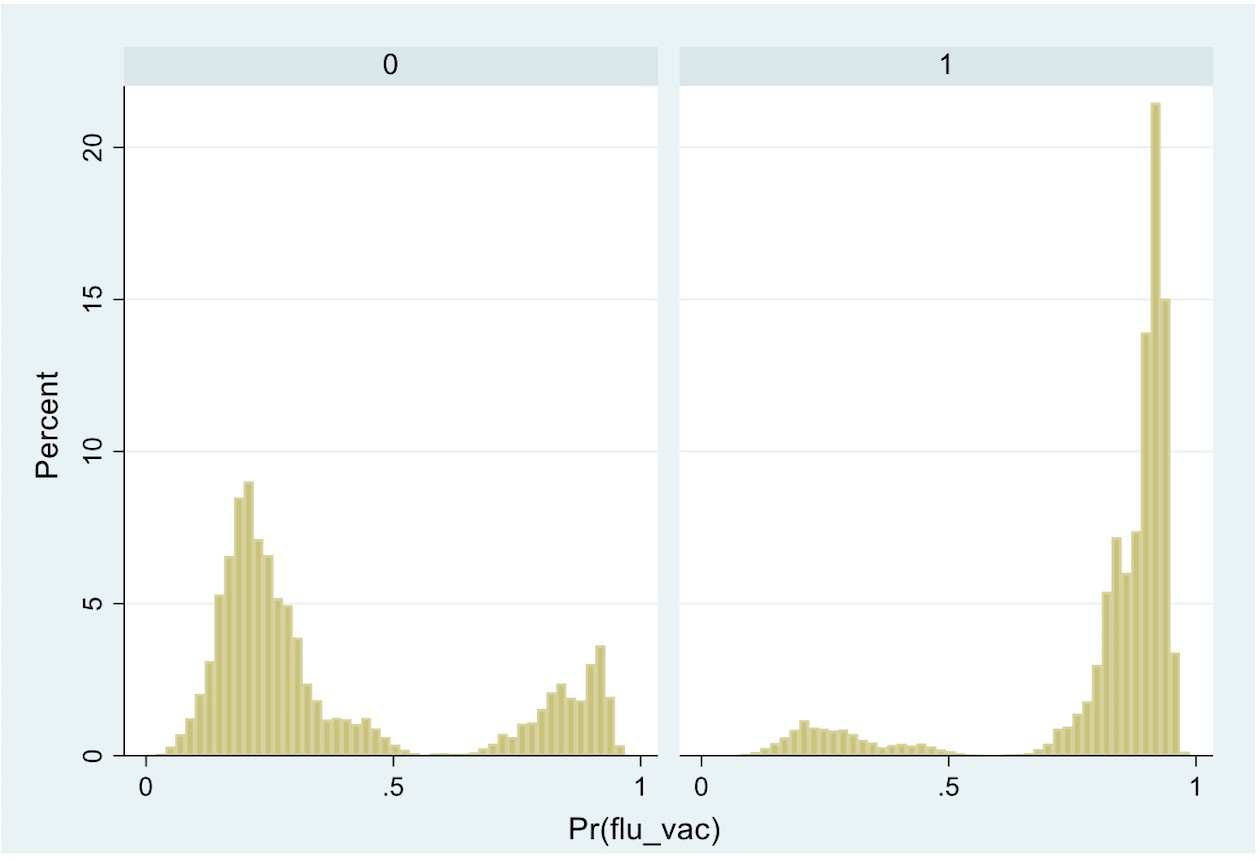

Supplement: keaa078_Supplementary_Data [file keaa078_supplementary_data.zip › keaa078-suppl_data/rhe-19-2088-File003.jpg]
